# Structural basis for oxygen degradation domain selectivity of the HIF prolyl hydroxylases

**DOI:** 10.1038/ncomms12673

**Published:** 2016-08-26

**Authors:** Rasheduzzaman Chowdhury, Ivanhoe K. H. Leung, Ya-Min Tian, Martine I. Abboud, Wei Ge, Carmen Domene, François-Xavier Cantrelle, Isabelle Landrieu, Adam P. Hardy, Christopher W. Pugh, Peter J. Ratcliffe, Timothy D. W. Claridge, Christopher J. Schofield

**Affiliations:** 1 Chemistry Research Laboratory, Department of Chemistry, Oxford Centre for Integrative Systems Biology, University of Oxford, Mansfield Road, Oxford OX1 3TA, UK; 2 Nuffield Department of Clinical Medicine, University of Oxford, Henry Wellcome Building for Molecular Physiology, Roosevelt Drive, Oxford OX3 7BN, UK; 3 UMR8576 CNRS-Lille University, Villeneuve d'Ascq 59655, France; 4 Ludwig Institute for Cancer Research, University of Oxford, Roosevelt Drive, Oxford OX3 7DQ, UK

## Abstract

The response to hypoxia in animals involves the expression of multiple genes regulated by the αβ-hypoxia-inducible transcription factors (HIFs). The hypoxia-sensing mechanism involves oxygen limited hydroxylation of prolyl residues in the N- and C-terminal oxygen-dependent degradation domains (NODD and CODD) of HIFα isoforms, as catalysed by prolyl hydroxylases (PHD 1–3). Prolyl hydroxylation promotes binding of HIFα to the von Hippel–Lindau protein (VHL)–elongin B/C complex, thus signalling for proteosomal degradation of HIFα. We reveal that certain PHD2 variants linked to familial erythrocytosis and cancer are highly selective for CODD or NODD. Crystalline and solution state studies coupled to kinetic and cellular analyses reveal how wild-type and variant PHDs achieve ODD selectivity via different dynamic interactions involving loop and C-terminal regions. The results inform on how HIF target gene selectivity is achieved and will be of use in developing selective PHD inhibitors.

## Abstract (web summary)

The response to hypoxia involves multiple genes regulated by the hypoxia inducible transcription factors (HIFs), whose stability is regulated by prolyl hydroxylation. Here the authors provide a molecular basis for the substrate selectivity of the HIF prolyl hydroxylases that can be altered in erythrocytosis and cancer.

## Graphical abstract

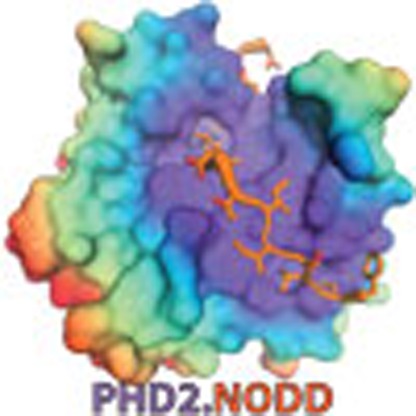

Animals respond to limiting oxygen availability by the context-dependent upregulation of the expression of multiple genes as promoted by increased levels of the α-subunit of the αβ-heterodimeric hypoxia-inducible factors (HIFs). The ferrous iron and 2-oxoglutarate (2OG)-dependent HIF prolyl hydroxylases (PHDs/EGLNs) catalyse *trans*-4-prolyl hydroxylations on HIFα subunits (Fig. 1), thus promoting their binding to the von Hippel–Lindau protein (VHL), a targeting component of a ubiquitin E3 ligase, and signalling for HIFα degradation under normoxic conditions^1^,^2^,^3^,^4^,^5^. In hypoxia, HIFα escapes VHL-mediated degradation, translocates into the nucleus, where it dimerizes with HIFβ to form the functional HIF complex that activates the transcription of gene arrays. Evidence from both biochemical and genetic studies support the roles for the PHDs as the most important identified hypoxia sensors for the HIF system. As HIF target genes include those encoding for erythropoietin, vascular endothelial growth factor and many medicinally important proteins, therapeutic manipulation of the HIF system, in particular by PHD inhibition, is of interest. PHD inhibitors are in clinical trials for anaemia treatment via HIF-mediated erythropoietin upregulation^6^,^7^, although these inhibitors are not PHD isoform selective.

Bioinformatics imply that the HIF–PHD–VHL triad-based hypoxia-sensing system is likely to be present in almost all animals, but not in other eukaryotes^8^,^9^. In early animals (that is, those that emerged before the whole genome duplications), there is normally one copy of the PHDs and one of HIFα with only one HIFα hydroxylation site. However, concomitant with the evolution of complex animals and associated whole genome duplications, animals with multiple copies of HIFα/PHDs emerged. The increases in the complexity of the HIF system probably reflect the needs for more sophisticated hypoxia response mechanisms involving the complex cardiovascular systems present in higher animals. Humans have three HIFα isoforms (with HIF-1α/-2α being the best characterized) and three PHDs. The PHD-catalysed hydroxylations of HIF-1α/HIF-2α in higher animals occur in amino- and carboxy-terminal oxygen-dependent degradation domains (NODD and CODD)^10^,^11^,^12^. Non-HIF substrates for the PHDs have been reported but the physiological significance of these in the hypoxic response is presently unclear (for review, see ref. 13).

Different roles for the PHDs and HIFα isoforms including for their ODDs are emerging. NODD hydroxylation is more sensitive to hypoxia than CODD^14^,^15^. The three human PHDs exhibit different NODD/CODD selectivities, with PHD3 being substantially more selective for CODD than PHD1/2 (refs 16, 17 and Supplementary Fig. 1). Some clinically observed heterozygous mutations to PHD2 (which genetic studies have revealed is indispensable in mice^18^,^19^,^20^, unlike PHD1 and PHD3), stabilize HIFα and cause familial erythrocytosis^21^ or are linked to cancer^22^. We found that certain PHD2 variants linked to familial erythrocytosis and cancer are highly selective for CODD or NODD. We therefore worked to obtain structural information on how the different ODDs bind to the PHDs, focusing on PHD2. Here we report studies on the molecular basis for ODD selectivity of the wild-type (wt) and variant PHDs.

## Results

### PHD2 clinical variants are selective for CODD or NODD

**Figure 2:**
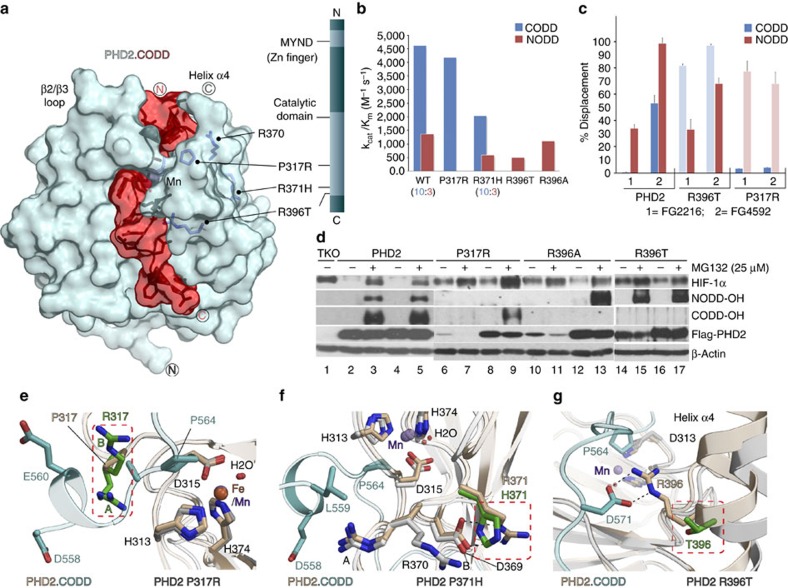
Clinically observed variants in PHD2 have altered selectivities. (**a**) View from the PHD2.CODD complex (PDB: 3HQR) showing locations of PHD2 clinical variants with altered ODD selectivities. (**b**) Kinetic analyses show the P317R and R396X variants are highly selective for CODD and NODD, respectively; R371H is less efficient at the same CODD/NODD activity ratio (10:3) as wt PHD2 with (almost) unaltered selectivity. *k*_cat_/*K*_m_ values are calculated from the average *k*_cat_ and *K*_m_ values (Supplementary Table 3). (**c**) One-dimensional ^13^C-selective clean in-phase (CLIP)–HSQC NMR reveals apparently selective displacement of NODD/CODD from PHD2 wt/clinical variant complexes by using PHD inhibitors (FG2216/ FG4592) (Supplementary Fig. 13). *n*=5 for wt and 2 for variants. (**d**) Selectivity studies using hydroxy-proline antibodies (NODD-OH and CODD-OH) and PHD 1–3 TKO MEF cells. MG 132 was used to block proteasomal degradation. In TKO cells, HIF-1α is not hydroxylated (lane 1); both NODD/CODD are fully hydroxylated in cells expressing wt PHD2 (lanes 3 and 5). Highly selective NODD/CODD hydroxylation is observed with variant PHDs irrespective of expression level of the Flag-tagged proteins. (**e**,**f**,**g**) Views from PHD2 P317R, R371H and R396T crystal structures superimposed with PHD2.CODD complex, suggesting substantial impact of the substitutions on substrate binding.

Analysis of a PHD2.*N*-oxalyl glycine (NOG).CODD complex structure^23^ led us to propose that certain erythrocytosis-associated PHD2 variants (P317R^24^,^25^ and R371H^26^) would have altered substrate selectivities (Fig. 2a). Indeed, recombinant P317R PHD2 retains full CODD activity compared with wt PHD2, but strikingly does not hydroxylate NODD (Fig. 2b), suggesting its phenotype could be associated with loss of NODD hydroxylation. R371H PHD2 retains >60% NODD activity and is equally active as wt PHD2 with CODD in endpoint assays (Supplementary Tables 1 and 2); kinetic analyses show R371H is less efficient compared with wt PHD2 (for R371H, *k*_cat_/*K*_m_ is reduced by ∼50% for NODD and CODD; Fig. 2b). A PHD2 R396T variant, present in breast carcinoma^27^, and an R396A variant efficiently hydroxylate NODD, but both manifest loss of CODD activity (Fig. 2b). To investigate their selectivities in cells, the PHD2 variants were expressed in mouse embryonic fibroblasts (MEFs) lacking all three PHDs (‘triple knockout'/TKO MEFs deficient in HIF hydroxylation). Using hydroxy-NODD/CODD-specific antibodies^15^, we observed high NODD/CODD selectivities for the variants consistent with the isolated protein results (Fig. 2d); both sites were efficiently hydroxylated with wt PHD2.

We then investigated the structural basis of the striking differences in the NODD/CODD selectivities of the clinical PHD2 variants. Structures of the P317R, R371H and R396T variants with a clinically used inhibitor^6^,^28^ reveal similar overall folds to wt PHD2, that is, a modified double-stranded-β-helix (DSBH) core (β-strands I–VIII), supporting 2OG and His-X-Asp…His-mediated Fe(II) binding. The structures suggested that these variants probably manifest altered substrate binding (Fig. 2e–g), but do not explain how their differing NODD/CODD selectivities arise. The PHD2.NOG.CODD structure reveals elements involved in CODD binding including a ‘β2/β3 loop'^23^,^29^ but there is no information on the details of NODD binding, nor on how ODD selectivity is achieved. We therefore pursued biophysical and biochemical analyses on PHD2.NODD complexes.

### PHD2.ODD complexes reveal insights into ODD selectivities

We obtained a PHD2.2OG.CODD structure using the strategy used for the PHD2.NOG.CODD complex^23^, but did not obtain PHD2.NODD crystals using this procedure, possibly because NODD binds PHD2 less tightly, as indicated by a lower dissociation rate constant (*k*_d_) for CODD compared with NODD^29^. We then used a ‘disulfide cross-linking' strategy^30^,^31^ to form stable PHD2.Mn.NOG.NODD complexes, where PHD2_QM1_ (C201A/P317C/R281C/R398A) or PHD2_QM2_ (C201A/V314C/R281C/R398A) and NODD_DC_ (L397C/D412C) are cross-linked via appropriately positioned cysteines (Supplementary Fig. 2). Molecular dynamics (MD) simulations predict the PHD2.Fe.2OG.NODD solution structure is very similar to that of the disulfide-linked complex, PHD2_QM1_.Mn.NOG.NODD_DC_ (or PHD2.NODD) (Supplementary Fig. 3).

**Figure 3:**
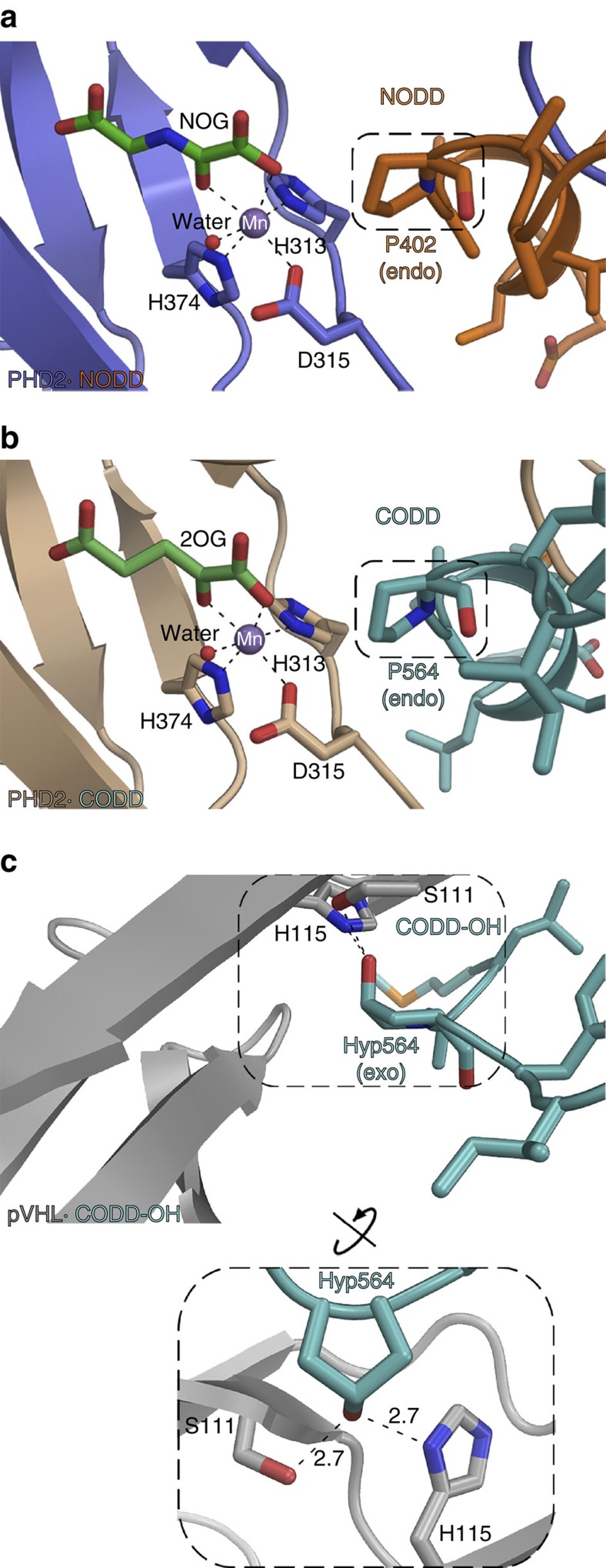
PHD active-site chemistry. Binding of proline (NODD/CODD) to PHD2 and hydroxyproline/Hyp (CODD-OH) to the VHL component of the VCB complex. (**a**,**b**) Conserved binding modes of the Pro402_NODD_/Pro564_CODD_ to PHD2 (PDB: 5L9V and 5L9B). It is noteworthy that the proline C4-methylene adopts the *endo*-conformation when bound to PHD2 in both the NODD and CODD complex structures. (**c**) In contrast, Hyp564_CODD-OH_ adopts the *exo*- conformation when bound to the VCB complex (PDB: 1LM8)^32^,^33^,^34^. Binding of O_2_ is proposed to be limiting in PHD-ODD catalysis^57^,^58^. It is noteworthy that the metal bound water, which is replaced by O_2_ in catalysis, is similarly positioned in both the NODD and CODD complexes (with NOG/Mn substituted for 2OG/Fe).

**Figure 4:**
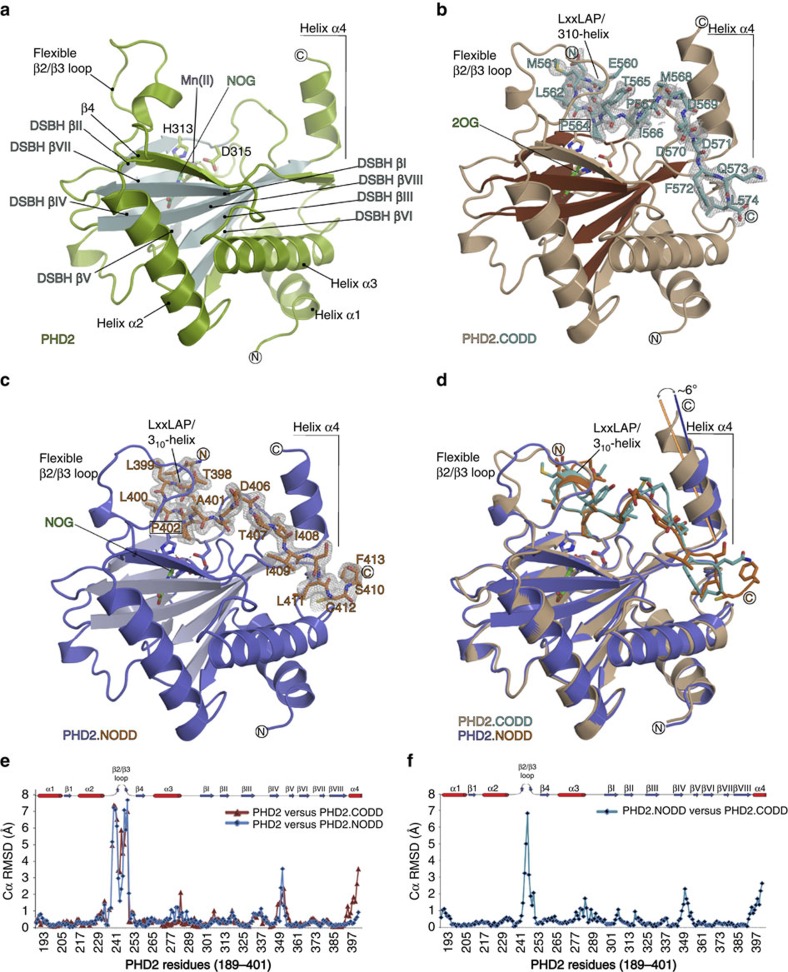
Overall binding modes of NODD and CODD to PHD2. Views from PHD2.NOG (**a**, PDB: 5L9R), PHD2.2OG.CODD (**b**, PDB: 5L9B) and PHD2.NOG.NODD complexes (**c**, PDB: 5L9V) showing secondary structural elements involved in NODD/CODD selectivity. (**d**) Superimposition of PHD2.CODD and PHD2.NODD structures reveals apparently more ‘induced fit' in CODD (compared with NODD) binding involving the β2/β3 loop and α4. (**e**) Cα r.m.s.d. plots of PHD2 structures with/without CODD (brown) and NODD (blue) reveal similar PHD2 backbone conformations for the major and minor β-sheets of the DSBH and surrounding three α-helices (1–3), but clear differences especially in the β2/β3 loop (aa 237–250), βIV/βV loop (aa 348–353) and C-terminal helix α4 (393–401, CODD). (**f**) Cα r.m.s.d. plot comparing the PHD2.NODD and PHD2.CODD structures shows differences mainly in the β2/β3 loop (aa 243–250) and C-terminal regions (aa 393–401).

Both ODDs bind to PHD2 in an extended form (Cα root mean square deviation (r.m.s.d.), CODD_558–574_/NODD_396–412_, 2.4 Å) with several bends and an N-terminal 3_10_-helix containing the HIFα LXXLA**P**^3^,^5^ motif. The target prolines (P402/NODD and P564/CODD, both in C^4^ *endo* conformation^32^) are located at the apexes of the bends and, similar to metal and 2OG, are deeply embedded in the active site (Fig. 3). Comparison of the ODD complex structures and those without substrate reveals clear differences in NODD/CODD binding, especially in β2/β3 loop and C-terminal regions (Fig. 4).

**Figure 5:**
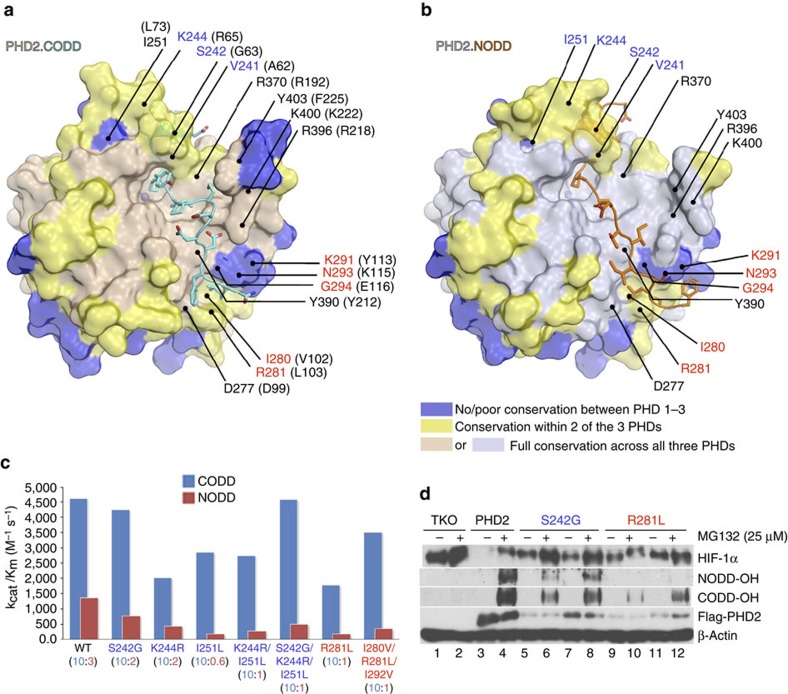
Combined biophysical and biochemical analyses identify NODD/CODD selectivity determinants in the PHDs. Surface representations of (**a**) PHD2.CODD and (**b**) PHD2.NODD complexes showing sequence differences that inform on selectivity determinants in addition to clinically observed variant sites (Pro317, Arg371 and Arg396) (Supplementary Fig. 1 gives a sequence alignment of PHDs). (**c**) Catalytic efficiencies (*k*_cat_/*K*_m_) of PHD2 wt and the variants for hydroxylating CODD and NODD; values in the parentheses are CODD/NODD activity ratios; selected variants were tested in cells (**d**). Assays of the indicated PHD2 variants in TKO cells as described in Fig. 2d.

Binding of residues N-terminal to target prolines involves the DSBH βII, βII–III loop, β2/β3 loop, βVI–VII loop, βVIII and βIII regions (Supplementary Fig. 4). Excepting the β2/β3 loop, these elements are well-conserved in animal PHDs. Notably, β2/β3 loop residues (Val241, Ser242, Lys244 and Ile251) interact with the ‘XX' residues of the LXXLAP motif, which differ in NODD and CODD (Glu560/Met561_CODD_; Thr398/Leu399_NODD_) (Supplementary Fig. 4). Substitution of β2/β3 loop Val241, Ser242, Lys244 and Ile251 residues to Ala, Gly, Arg and Leu, respectively, as present in the CODD-selective PHD isoform, PHD3 (Ala62_PHD3_, Gly63_PHD3_, Arg65_PHD3_ and Leu73_PHD3_) causes increased CODD selectivity, revealing specific β2/β3 elements contributing to selectivity (Fig. 5).

Binding of ODD residues C-terminal of the target prolines involves DSBH βVIII, βIII, helix α3 and the α3-βI loop (Supplementary Fig. 5). In addition, CODD, but not NODD, interacts with α4 including via salt-bridging (Asp571_CODD_–Arg396_PHD2_). NODD makes more hydrophobic contacts than CODD with the C-terminal region of PHD2 (Supplementary Fig. 5). Using biochemical and structural studies, we identified four more residues in α3 and α3-βI loop regions (PHD2/Ile280, Arg281, Ile292 and Gly294 that are substituted in PHD3/Val102, Leu103, Val114 and Glu116, respectively) and which play roles in determining selectivity (Fig. 5 and Supplementary Figs 6 and 7).

### NMR analyses of PHD2 interactions with ODDs

**Figure 6:**
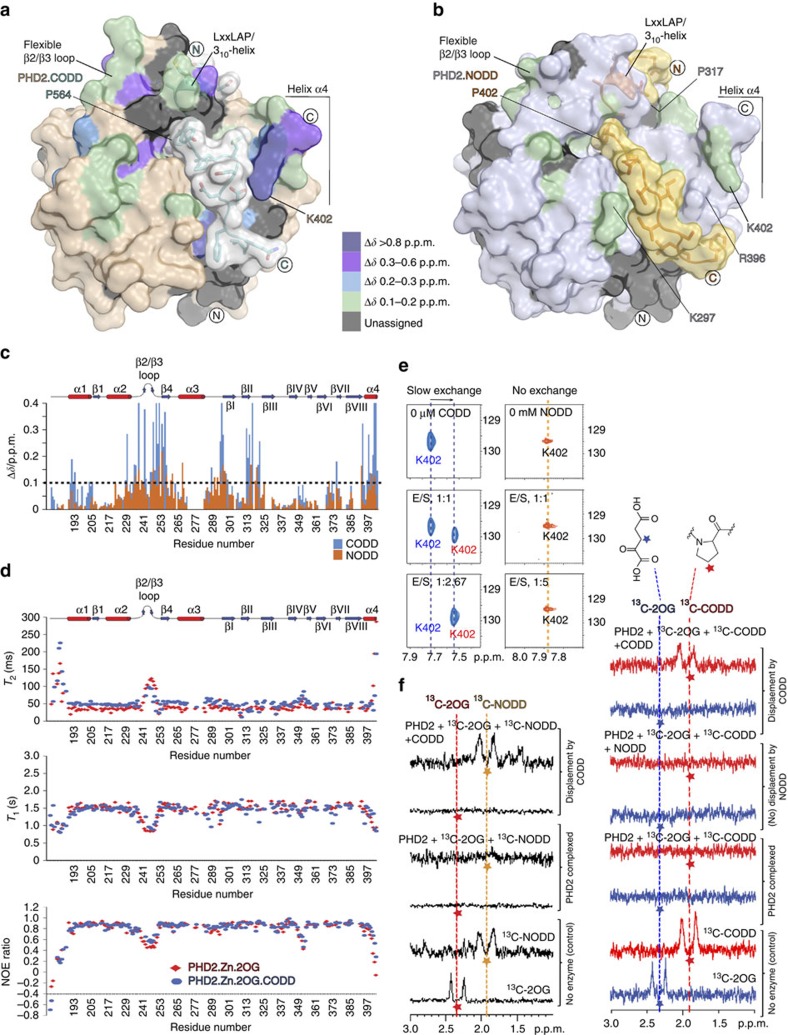
NMR studies reveal dynamics of ODD selectivity determinants in solution. Surface representations of PHD2.CODD (CODD in grey, PDB: 5L9B) (**a**) and PHD2.NODD (NODD in yellow, PDB: 5L9V) (**b**) showing perturbed regions (colour coded) on ODD binding with differences in chemical shift values in **c**. On CODD binding, changes concentrate in: N terminus (α1, aa 190–195; α2, 229–231; β2/β3 loop, 234–254; β4, 255–258; and α3-βI, 294–298), the DSBH core (βI, 299–300; βII, 312–317; and βII–βIII, 319–322) and C-terminal regions including the βVIII-α4 loop (391–393) and α4 (395–402). Compared with CODD, binding of NODD induces fewer changes including in: β2/β3 loop (236–241 and 247–255), β4 (257–258), β4-α3 (259–262), α3 (268), α3-βI (290–299) βII–βIII (318–319), and βVI–βVII (369–370), and smaller changes (relative to CODD) in α4 (398–400). (**d**) ^15^N relaxation (*T*_1_/*T*_2_) and ^1^H-^15^N NOE measurements reveal β2/β3 loop dynamics that are diminished on CODD binding. (**e**) ^15^N HSQC experiments show that CODD binding directly perturbs PHD2 C terminus as exemplified by slow-exchange titration behaviour of Lys402 (291K), whereas binding of NODD has less/no influence (310K). (**f**) Competitive binding experiments (one-dimensional ^13^C-selective clean in-phase (CLIP)–HSQC) reveal CODD binding displaces NODD but not vice versa within limits of detection. ^13^C-2OG/ ^13^C-NODD/ ^13^C-CODD-selective excitations are indicated by coloured asterisks.

We then used nuclear magnetic resonance (NMR) to investigate whether the crystallographically observed differences in ODD binding apply in solution. We first assigned backbone resonances of PHD2.Zn.2OG with and without CODD (using Zn^II^ as an Fe^II^ substitute, Supplementary Fig. 8). Consistent with the crystallographic results, large chemical shift perturbations manifested at the PHD2.CODD binding interface including in the β2/β3 loop region and α4 (Fig. 6a–e); ^15^N relaxation and heteronuclear NOE measurements reveal the dynamic nature of these regions, particularly the β2/β3 loop (Fig. 6d). Notably, the plasticity of the β2/β3 loop is substantially reduced on substrate binding (>30% with CODD as manifested by comparing the relative changes in the ^15^N *T*_2_ and ^1^H-^15^N NOE values between the PHD2. 2OG and PHD2.2OG.CODD complexes), revealing a role for the β2/β3 loop in stabilizing the PHD2.ODD complexes.

Similar to CODD, when NODD was titrated into labelled PHD2.Zn.2OG, chemical shift perturbations were observed at the binding interface, including in the β2/β3 loop (Fig. 6c). However, an important difference between NODD and CODD binding is that in contrast to CODD, substantially less perturbation was observed in the helix α4 with NODD (Fig. 6b,c and e), supporting the crystallography, that is, α4 adopts similar conformations whether bound to NODD or not (Fig. 4). To investigate NODD binding, we also used competition experiments with [^13^C]-labelled NODD and unlabelled CODD, and with [^13^C]-labelled CODD and unlabelled NODD. Consistent with the crystallographic analyses, the results reveal that CODD and NODD bind competitively with each other to PHD2.Zn.2OG with a clear preference for CODD (Fig. 6f).

### CODD-selective residues are conserved during PHD evolution

To investigate how ODD binding relates to selectivity, we produced PHD3 variants aimed at increasing the NODD activity of PHD3: R65K and L73I PHD3 variants manifest increased NODD hydroxylation relative to wt PHD3 (Supplementary Fig. 9). We also investigated conservation of PHD2 residues other than the clinical variants (see below) involved in selectivity (that is, Ser242, Lys244, Ile251, Ile280, Arg281, Ile292 and Gly294 residues) using structurally informed bioinformatics on animal PHDs; except for Ile251_PHD2_ (which is often a Leu as in PHD3), none of these residues are well conserved. Notably, kinetic studies reveal PHD2 I251L is remarkably (∼5-fold) selective for CODD over NODD (Fig. 5c), indicating there is a preference for conservation of CODD-selective residues during PHD evolution (see below).

### Structural analyses rationalize PHD2 variant selectivities

Comparison of the structures rationalizes the selectivities of the clinically observed P317R, R371H and R396T variants. Pro317_PHD2_ (βII-III loop) forms part of a hydrophobic patch binding the ODD LXXLAP-3_10_-helix. The P317R structure reveals Arg317_PHD2_ present in two conformations, one predicted to interact differently with the LXXLAP motif (Supplementary Fig. 10). The lack of reactivity of P317R PHD2 with NODD supports a relatively more important role for Pro317_PHD2_ in binding the LXXLAP residues in NODD than CODD.

Arg371_PHD2_ (βVI-VII loop) apparently positions the Arg370_PHD2_ side chain in ODD binding (Supplementary Fig. 11): Arg370_PHD2_ interacts with both Asp395_NODD_ (electrostatic) and Leu559_CODD_ (hydrophobic); substitution of Arg370_PHD2_ with alanine did not affect CODD hydroxylation, but gave ∼35% reduction in NODD activity, demonstrating Arg370–Arg371 is more important in NODD than in CODD hydroxylation.

In the PHD2.CODD structure, Arg396_PHD2_ (α4) is positioned to salt bridge with Asp571_CODD_; analysis of the ODD structures leads to the prediction that the R396T and R396A substitutions disrupt their interaction with Asp571_CODD_, but will not directly have an impact on NODD binding (Fig. 2g). Indeed, structures of R396T with/without NODD indicate how R396T substitution is tolerated for NODD binding (Supplementary Fig. 12). Interestingly, Asp571_CODD_ is also important in VHL–HIFα binding, where it salt bridges with Arg107_VHL_^33^,^34^; the high conservation of Arg396_PHD2_ and Arg107_VHL_ implies that both these interactions are important in PHD–VHL evolution^8^.

### PHD clinical inhibitors differently displace NODD or CODD

The revelation of differential contributions from specific PHD regions in ODD binding raises the possibility of identifying inhibitors that compete differently with NODD or CODD. Indeed, we found that FG2216 (IOX3)^35^, used in clinical trials for treatment of anaemia, preferentially displaces NODD over CODD from PHD2 (Fig. 2c). Analysis of PHD2.FG2216 complex structures, with and without CODD (PDB: 3HQU, 4BQX), suggests this is because of the relatively greater role of the LXXLAP region in NODD compared with CODD binding (with CODD, loss of LXXLAP binding is compensated by interactions involving C-terminal regions). In contrast, FG4592 (Roxadustat)^35^, a newer compound in clinical trials^36^, efficiently displaces both CODD and NODD from PHD2 likely to be due to its phenoxy group, which projects into regions binding the residues C-terminal to the hydroxylated prolines of both ODDs (Supplementary Fig. 13). Notably, the selectivity of NODD/CODD displacement by the inhibitors differs for the R396T and P317R variants compared with wt PHD2. In the case of R396T (which does not hydroxylate CODD efficiently, 2b), both FG2216 and FG4592 manifest NODD displacement less than wt PHD2 (Fig. 2c). P317R (which does not hydroxylate NODD, 2b) differs in that both inhibitors displace CODD to <10%, which is also less than for wt (with FG4592). These results imply development of inhibitors differentially blocking NODD versus CODD binding should be possible.

## Discussion

PHD variant-associated erythrocytosis has been near exclusively linked to PHD2 (ref. 21). Our finding that certain clinically observed PHD2 mutations substantially alter ODD selectivity, that is, the erythrocytosis-associated P317R PHD2 (refs 24, 25) variant towards CODD, and the breast cancer-associated R396T PHD2 (ref. 27) variant towards NODD, implies that altered PHD2 selectivity may have pathological consequences. Biophysical analyses employing crystallography and NMR reveal that the molecular basis of PHD isoform and variant selectivity involves enzyme–substrate interactions involving β2/β3 loop residues (that bind the LXXLAP motif) and helices α3 and α4, regions which display sequence variations between the PHD isoforms. The results reveal higher conservation of residues correlating with maintenance of CODD over NODD selectivity during the course of animal evolution that ‘CODD-type' hydroxylation probably evolved before that of NODD^8^, the greater importance of induced fit in CODD compared with NODD binding and clinical/genetic evidence that loss of wt PHD2 cannot be compensated for by PHD 1/3 (refs 19, 20, 37, 38, 39). The identification of specific interactions determining PHD selectivity for ODDs opens the way forward to PHD isoform and ODD selective inhibitors, which may be useful for the upregulation of specific sets of HIF target genes. The identification of the features determining HIFα ODD selectivity should also be of interest with respect to validating reports of non-HIF substrates for the PHDs.

## Methods

### Materials

Chemicals were from Sigma-Aldrich, Merck Chemicals and Alfa Aesar. Isotopically labelled compounds for NMR were from Apollo Scientific (Stockport, UK), Cambridge Isotope Laboratories (Tewksbury, MA, USA), Cortecnet (Voisins-Le-Bretonneux, France) or Euriso-Top (Paris, France). Matrix-assisted laser desorption/ionization–time of flight matrices, matrix buffers and calibrants were from LaserBio Labs (Valbonne, France), HIF-1α NODD_395–413_ and HIF-1α CODD_556–574_ peptide substrates (C-terminal amides) were from GL Biochem (Shanghai, China) and DNA primers were from Sigma Genosys.

### Recombinant protein production

DNA sequences encoding human PHD3 and truncated PHD2_181–426_ (PHD2) were cloned into pET-28a(+)/pET-24a(+) by using restriction enzymes NheI/ BamHI, to enable production of proteins with/without an N-terminal His_6_ tag^23^. The His_6_ tags were removed by proteolysis using thrombin, which leaves a flanking sequence of GSHMAS, N terminus to the PHD2 wt and variant sequences. The PHD2 and PHD3 variants were prepared using site-directed mutagenesis (Stratagene). For the production of isotopically labelled PHD2, a construct encoding for PHD2_181–402_ was cloned into the pET-28a(+) vector. All constructs were verified by DNA sequencing.

PHD2, PHD3 and their variants were produced in *Escherichia coli* BL21(DE3) cells by induction with 0.5 mM isopropyl β-D-1-thiogalactopyranoside for 4–6 h at 28 °C/37 °C. Cells were freeze–thawed and lysed in 20 mM Tris·HCl pH 7.0–7.5, 0.5 M NaCl and 5% glycerol (or alternatively in 0.1 M MES·Na pH 5.8) by sonication. Proteins were purified by Ni^2+^ affinity (tetracarboxymethyl ethylenediamine) or by cation-exchange (SP Sepharose Fast Flow, GE Healthcare) chromatography followed by size-exclusion chromatography (0.1 M Tris·HCl pH 7.5, 0.1 M NaCl/PHD2 and 50 mM TriṡHCl pH 7.5, 0.5 M NaCl, 5% glycerol and 0.5 mM tris(2-carboxyethyl)phosphine/PHD3). PHD2 wt and variants were exchanged into 50 mM Tris·HCl buffer pH 7.5 and stored at 25–30 mg ml^−1^. PHD3 proteins were buffer exchanged into 50 mM TriṡHCl pH 7.5, 0.5 M NaCl, 5% glycerol and 0.5 mM tris(2-carboxyethyl)phosphine (TCEP) and stored at 2–5 mg ml^−1^. Protein purity was assessed by SDS–PAGE; proteins were characterized by electrospray ionization mass spectrometric analyses under non-denaturing and/or denaturing conditions.

Isotopically labelled PHD2_181–402_ was produced in *E. coli* BL21(DE3). Cells were grown at 37/30 °C (to an OD_600_ of 0.6–1.0) either in 600 ml of M9 minimal media supplemented with 1 g l^−1^ of ^15^N-labelled NH_4_Cl and 10 g l^−1^ D-glucose (for ^15^N*-labelled PHD2*_*181–402*_) or in 600 ml of M9 minimal media in D_2_O supplemented with 1 g l^−1^ of ^15^N-labelled NH_4_Cl and 4 g l^−1^ of ^2^H,^13^C-labelled D-glucose (for ^*2*^*H,*^*13*^*C,*^*15*^*N-labelled PHD2*_*181–402*_). Protein production was induced with 0.2 mM isopropyl β-D-1-thiogalactopyranoside (overnight, 28/18 °C). Cells were resuspended and lysed in buffer containing 50 mM Hepes-Na, 0.5 M NaCl and 5 mM imidazole (pH 7.8). The labelled proteins were purified by gravity-flow Ni^2+^ affinity (nitrilotriacetic acid) chromatography. *Apo*-PHD2_181–402_ was produced by incubation (at 1 mg ml^−1^ protein concentration) with EDTA (0.2 M) in 15 mM ammonium acetate (pH 7.5) overnight at 4 °C. Protein purity was assessed by SDS–PAGE. The purified proteins were stored in 50 mM Tris-D11 pH 6.6 and 0.02% NaN_3_ at −80 °C.

### Enzyme assays

2OG turnover was measured by assaying [^14^C]-CO_2_ production^23^. Peptide hydroxylation was measured by matrix-assisted laser desorption/ionization–time of flight mass spectrometry with appropriate controls^23^. For initial velocity measurements, assays were carried out using at least two optimum time points (where the initial rate was linear) over a range of substrate concentrations (5–150 μM). Data were analysed using Graphpad Prism 5 for fitting into Michaelis–Menten equation that enabled kinetic parameters, *K*_m_ (or apparent *K*_m_) and *k*_cat_ to be determined by using nonlinear regression, least squares fitting ±95% confidence intervals. *k*_cat_/*K*_m_ values are calculated from the mean *k*_cat_ and *K*_m_ values for wt and variant enzymes.

### Cell-based studies

The effects of PHD variants on HIF hydroxylation were assayed in cells with a PHD-null background using hydroxy-proline-specific antibodies^15^. The TKO PHD1–3 null MEFs (TKO MEFs) were generated from Phd^1−/−^; Phd2^fl/−^; Phd3^−/−^ embryos derived by appropriate mouse intercrosses, followed by inactivation of the remaining Phd2 allele by adenovirus expressed Cre-recombinase in cell culture^40^. Before further experiments, the TKO MEFs were validated to manifest lack of HIF-1α prolyl hydroxylation by immunoblotting using hydroxylation site-specific antibodies as previously described^15^. Constructs encoding for 3 × Flag-tagged PHD2 in pcDNA3 were used to generate NODD/CODD-selective variants (S242G, R281L, P317R, R396T and R396A) by site-directed mutagenesis (Promega) and subcloned into pRRL lentiviral vector. PHD2 virus were produced in 293T cells by transient transfection for 48 h. Wt and variant PHD2 were re-expressed in the TKO MEFs by viral infection for 44 h, followed by 4 h incubation with 25 μM MG 132, to block proteasomal degradation; this procedure enabled accumulation and simultaneous measurement of both hydroxylated CODD and NODD even in the presence of oxygen. The cells were then lysed in urea/SDS buffer (6.7 M urea, 10 mM Tris-HCl pH 6.8, 10% glycerol, 1% SDS and 1 mM dithiothreitol). Hydroxylation of HIF-1α was analysed by immunoblotting that employed antibodies specific for mouse HIF1α hydroxy prolines for both NODD (Hyp402) and CODD (Hyp577 analogous to Hyp564 in human HIF-1α) sites.

### NMR analyses

Data collection and sample details are given in Supplementary Table 4. Triple resonance experiments including HNCO, HN(CA)CO, HN(CO)CA, HNCA, HN(CO)CACB and HNCACB were conducted for PHD2 backbone assignment. Overall, 86% of backbone amide signals (excluding prolines) were assigned for ^2^H,^13^C,^15^N-PHD2_181–402_.Zn(II).2OG and 81% (excluding prolines) for ^2^H,^13^C,^15^N-PHD2_181–402_.Zn(II).2OG.CODD. Owing to the limited NODD solubility, triple resonance experiments were not performed with ^2^H,^13^C,^15^N-PHD2_181–402_.Zn(II).2OG.NODD.

Changes in amide chemical shifts (Δ*δ*, in p.p.m.) between ^2^H,^13^C,^15^N-PHD2_181–402_.Zn(II).2OG and ^2^H,^13^C,^15^N-PHD2_181–402_.Zn(II).2OG.CODD were measured using the formula^41^: 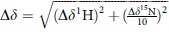. For PHD2 residues not assigned in CODD bound and/or unbound complexes, Δ*δ* were defined as 0 p.p.m. In cases where multiple peaks were assigned to the same residues, only the main peaks were considered for Δ*δ* measurements. To measure the chemical shift perturbation on NODD binding, ^15^N-HSQC spectra were recorded on the ^15^N-PHD2.Zn(II).2OG.NODD complex (Supplementary Table 4). The ‘transfer' of assignments for ^1^H and ^15^N chemical shifts between ^2^H,^13^C,^15^N-labelled and ^15^N-labelled proteins was carried out manually. Backbone amides for ^2^H,^13^C,^15^N-PHD2_181–402_.Zn(II).2OG.NODD were not assigned. Instead, the minimal shift assumption^42^,^43^, in which residues involved in NODD binding were mapped to the closest neighbouring peak, was applied for measuring the chemical shift changes.

^15^N relaxation (*T*_1_ and *T*_2_) and heteronuclear NOE experiments were carried out to investigate PHD2 dynamics (Supplementary Table 4). Ten delays were used for *T*_1_ measurements (0.02, 0.1, 0.2, 0.3, 0.4, 0.6, 0.7, 0.8, 1.0 and 1.5 s) and 11 delays for *T*_2_ measurements (4.24, 8.48, 16.96, 21.2, 25.44, 33.92, 42.4, 59.36, 76.32, 93.28 and 110.24 ms).

The clean in-phase–heteronuclear single-quantum correlation (HSQC) sequence was used in one-dimensional HSQC experiments without ^13^C decoupling for displacement measurements. Typical experimental parameters were as follows: acquisition time, 0.58 s; relaxation delay, 2 s; and number of transients, 256−1,600. The ^1^*J*_CH_ delay was set for 145 or 160 Hz. A 6.8 ms Q3 180° pulse was used and ^13^C selective irradiation was applied at 30.5 p.p.m. (2OG C-4) or 24.65 p.p.m. (proline C-4). The percentage displacement was measured based on the integrated area observed on addition of a competitor. A 100% displacement corresponds to a peak intensity similar to the one observed with the free label control, in the absence of protein. Percentage displacement was calculated according to equation: (*I*–*I*_0_)/(*I*_blank_–*I*_0_) where *I*_0_ is the intensity of the reporter in the presence of protein without inhibitor, *I* is the intensity of the reporter in the presence of protein and inhibitor, and *I*_blank_ is the intensity of the reporter without protein or inhibitor. ^13^C-2OG was labelled at carbon positions 1, 2, 3 and 4, and ^13^C-CODD/NODD was uniformly labelled at all carbon atoms of its proline ring. Solutions were buffered using Tris-D_11_ 50 mM (pH 7.5) dissolved in H_2_O-D_2_O (9:1). Assays were conducted at 298 K in solutions typically containing 50 μM apo-PHD2, 400 μM Zn(II), 50 μM labelled 2OG or CODD/NODD (where appropriate) and 800 μM competitor.

### Crystallography

PHD2 wt/variant complex crystals were grown as described in Supplementary Table 5. In general, crystals were cryoprotected by transferring to a solution of mother liquor supplemented with 25–30% (v/v) glycerol before being cryo-cooled in liquid N_2_. As described in Supplementary Tables 6–8, data were collected at 100 K using synchrotron radiation at the Diamond Light Source beamlines. Data were processed as outlined in Supplementary Tables 6–8.

Structures were solved by molecular replacement using PHASER^44^ (search model PDB ID 4BQX or 3HQR) and refined by alternative cycles of PHENIX^45^, CNS^46^ and BUSTER^47^ using the maximum-likelihood function and bulk-solvent modelling. Iterative cycles of model building in COOT^48^ and refinement proceeded until the *R*_cryst_/*R*_free_ values converged. Final rounds of refinement were performed by PHENIX^45^. MOLPROBITY^49^ were used to monitor the geometric quality of the models between refinement cycles and identify poorly modelled areas needing attention. Water molecules were added to peaks >1.8*σ* in 2*F*_o_–*F*_c_ electron density maps that were within hydrogen bonding distance to protein with reasonable hydrogen bonding geometry.

### MD simulations studies

Models for PHD2.Fe.2OG.HIF-1αNODD_395–412_, PHD3.Fe.2OG.HIF-1αNODD_395–412_ and PHD3.Fe.2OG.HIF-1αCODD_558–574_ were generated using crystal structures of PHD2_QM1_.Mn.NOG.HIF-1αNODD_DC(395–413)_ (or PHD2.NODD) and PHD2.Mn.2OG.HIF-1αCODD_558–574_ (or PHD2.CODD) as templates in the automodel feature of MODELLER v8.1 (ref. 50). The substrate–complex crystal structures as well as the above models were analysed. Acetyl groups were added to the N-termini of PHD2/substrate peptides and the C-termini were amidated.

All residues were in their default protonation states; H atoms were added using the software psfgen of VMD2.6. Histidines were protonated at the delta position. All crystallographic waters were included in models. Systems were solvated with a box padding of 18 × 18 × 18 Å^3^ dimension. Counter-ions were added where appropriate, to achieve neutrality of the simulation systems. The drift from an initial model was used as a measure of the relative ‘stability' of a given structure in a simulation. The drift was measured as the time-dependent Cα atom r.m.s.d. from the initial model. The initial rise in Cα r.m.s.d. in the first 0.2–0.3 ns is common in such simulations and has been attributed to relaxation of the protein on its transfer to the solution environment and/or inaccuracies in the potential function.

MD simulations were performed using NAMD2.6 (ref. 51) and the CHARMM-22 all atom force field with CMAP corrections^52^ with the TIP3P model for waters^53^. A set of force field parameters for 2OG and NOG were developed. Standardized protocols based on the original development of the force field were followed, to ensure the transferability of the parameters. A 1 fs time step was used to integrate the equations of motion. Coordinates were saved every 1 ps. Long-range electrostatic interactions were treated by the particle mesh Ewald algorithm^54^; a 10 Å cutoff was used for van der Waals interactions. Langevin dynamics controlled the temperature at 25 °C, with a damping factor of 5 ps^−1^. The Nosé–Hoover Langevin pressure control^55^,^56^ was used to maintain a pressure of 1 bar (piston period was 200 fs and the damping time scale was 100 fs). Energy was first minimized by a steepest descent procedure (10,000 steps), followed by production runs of 6 to 15 ns of unconstrained dynamics in the NpT ensemble. Overall, this corresponds to a total simulation time of 122 ns. Each system was composed of ∼60,000 atoms.

### Statistical analysis

Each endpoint assay results are the mean of three independent experiments with error bars representing the s.e.m. For kinetic measurements, each experiment was carried out (at least) in technical triplicate (*n*=3–9). Two independent biological replicates were done for the results in Figs 2d and 5d.

### Data availability

Atomic coordinates and structure factors for the crystal structures are deposited in the protein databank under the accession numbers 5L9R, 5L9B, 5L9V, 5LA9, 5LAS, 5LAT, 5LB6, 5LBB, 5LBC, 5LBE and 5LBF. Chemical shifts of the ^2^H,^13^C,^15^N-PHD2.Zn(II).2OG and ^2^H,^13^C,^15^N-PHD2.Zn(II).2OG.CODD have been deposited in the Biological Magnetic Resonance Data Bank under accession codes 26741 and 26742, respectively. The additional data that support the findings of this study are available from the corresponding author upon request.

## Additional information

**How to cite this article:** Chowdhury, R. *et al*. Structural basis for oxygen degradation domain selectivity of the HIF prolyl hydroxylases. *Nat. Commun.* 7:12673 doi: 10.1038/ncomms12673 (2016).

## Supplementary Material

Supplementary Information Supplementary Figures 1-13, Supplementary Table 1-8 and Supplementary References

## Figures and Tables

**Figure 1:**
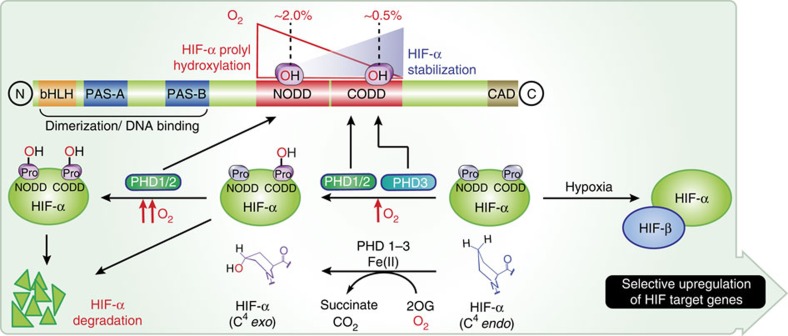
Overview of the HIF system. The figure shows the roles of HIFα NODD/CODD hydroxylation in the hypoxic response. Ordered ODD hydroxylation is tightly regulated in animals; NODD hydroxylation is more sensitive than CODD to hypoxia^14^,^15^. ODD hydroxylation significantly increases the affinity of hydroxylated HIFα for the VCB (VHL, elongins B and C) complex, thus signalling for HIFα degradation via proteosomal hydrolysis; the difference in *k*_d_ for hydroxylated versus non-hydroxylated CODD is ∼1,000-fold (33 nM versus 34 μM, respectively)^34^. In response to hypoxia, HIFα escapes ODD hydroxylation and forms the αβ-heterodimeric HIF complex that activates the transcription of a gene array.
