# Supplementary material for: Structural basis for oxygen degradation domain selectivity of the HIF prolyl hydroxylases
Source: Nat Commun. 2016 Aug 26;7:12673. doi: 10.1038/ncomms12673 (PMC5007464; doi:10.1038/ncomms12673)
Supplement: Supplementary Information — Supplementary Figures 1-13, Supplementary Table 1-8 and Supplementary References [file ncomms12673-s1.pdf]

SUPPLEMENTARY INFORMATION

Supplementary Figures

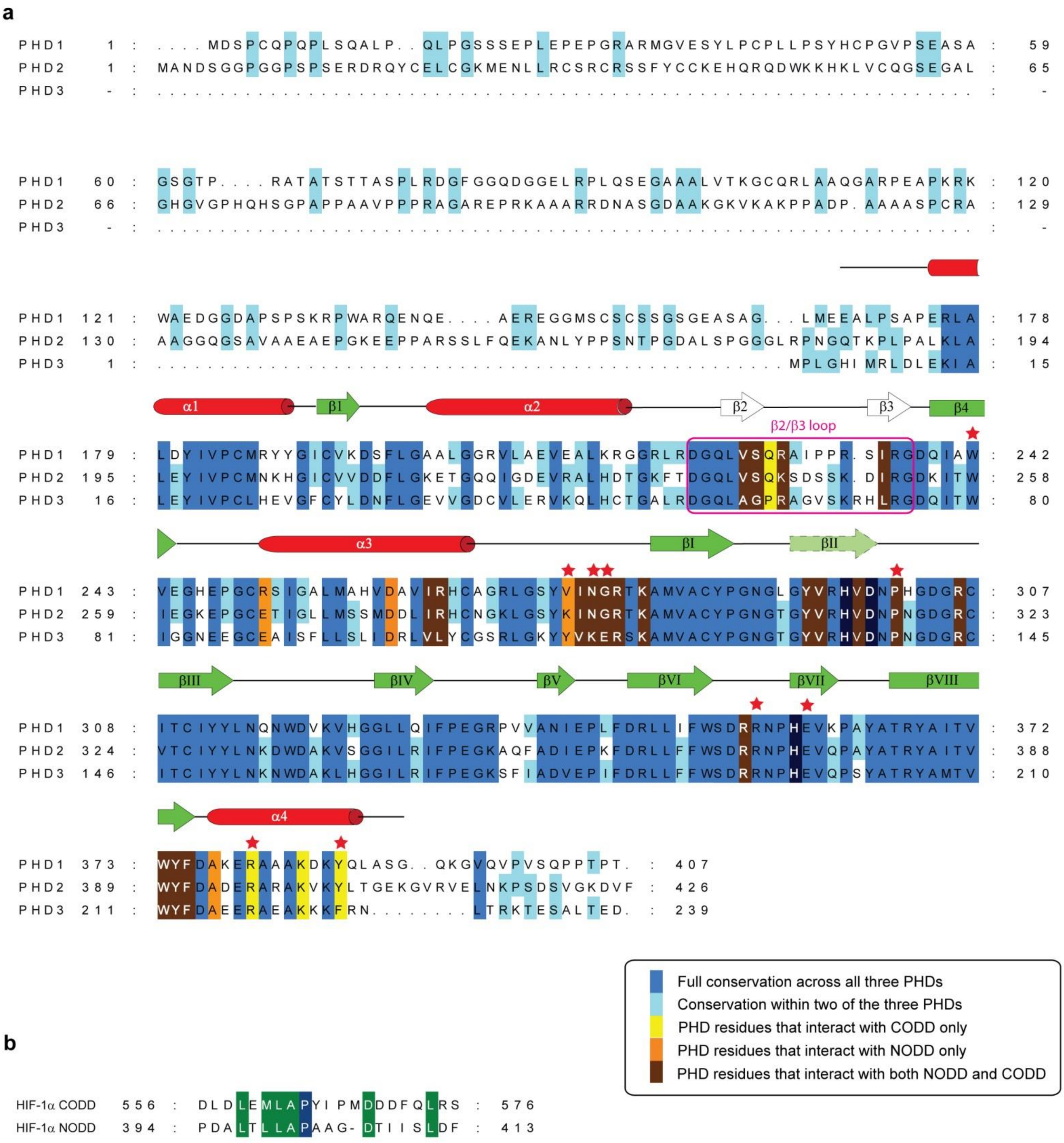

Supplementary Figure 1 | Sequence alignment of the three human PHD isoforms showing HIF-1α NODD/CODD substrate binding residues. (**a**) Sequence comparison of the human PHD isoforms (analyzed by ClustalW[1]). Secondary structures are in red (helices α1-4) and green (β1, β4 and DSBH βI- βVIII). Apart from their different *N*-terminal domains ($PHD1_{1-164}$/$PHD2_{1-180}$), conservation is substantially lost in two regions within the catalytic domains: the *C*-terminus and a flexible 'finger-like' loop between strands β2 and β3 (β2β3/loop, shown boxed in pink)[2,3]. PHD2 missense mutations as found in patients with erythrocytosis (K291I, P317R and R371H) and cancers (W258R, N293S, G294S, E375V, R396T and Y403C, COSMIC database[4]) are highlighted by red asterisks. (**b**) Structure-based alignment of HIF-1α NODD and CODD as observed bound in PHD2 complex structures.

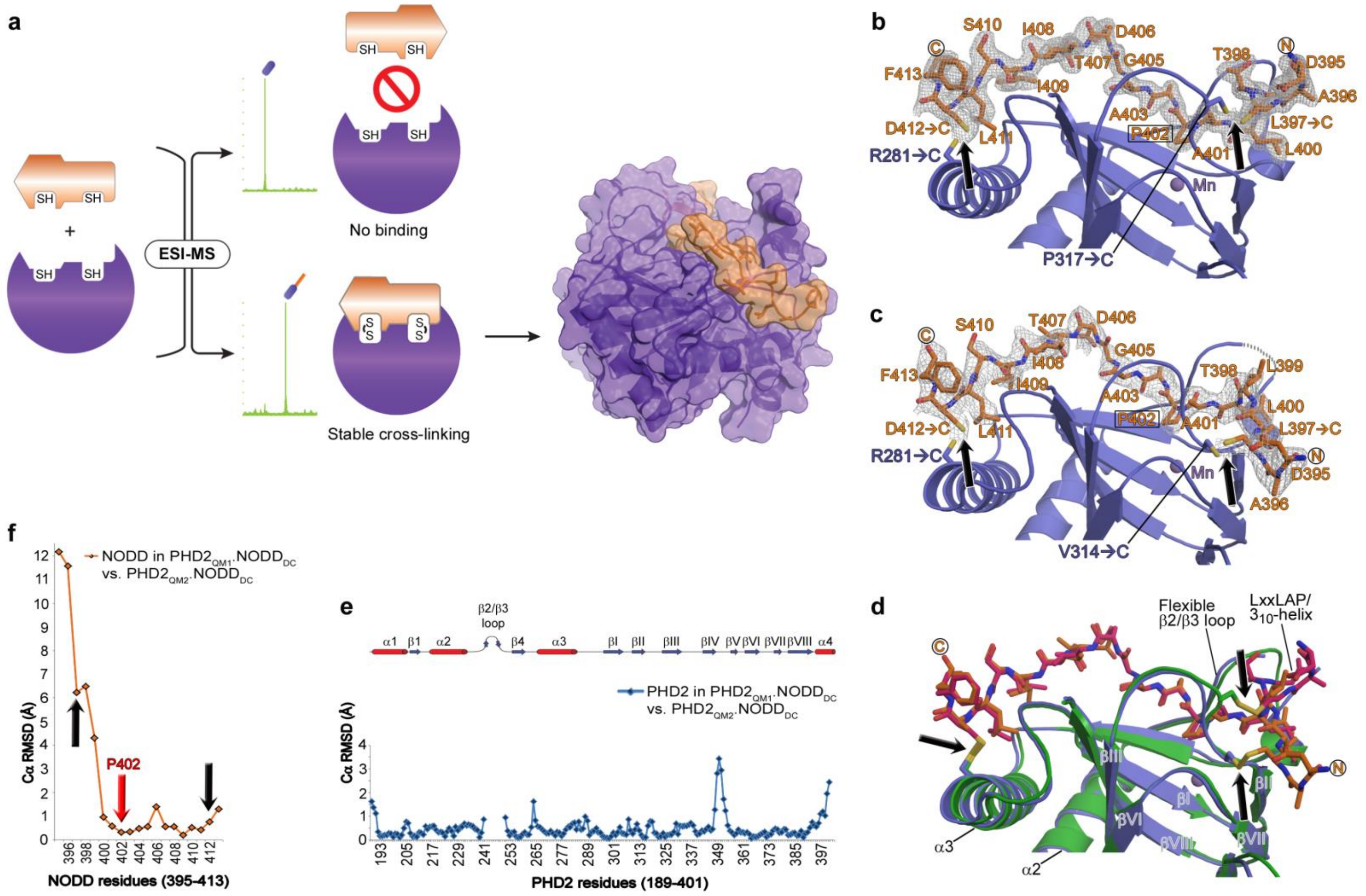

Supplementary Figure 2 | Crystallization of the PHD2.HIF1αNODD$_{395\text{-}412}$ complex. We attempted various approaches to obtain a PHD2.NODD structure including a 'mutation approach' aimed at blocking formation of a crystal form incapable of productive substrate binding, which we successfully employed to obtain a PHD2.Mn.NOG.HIF1αCODD$_{556\text{-}574}$ structure[2]. However, likely because PHD2 forms a less stable complex with NODD than CODD, these attempts were unsuccessful for the NODD. We then used an enzyme-substrate disulfide 'cross-linking' strategy[5,6], in combination with the 'mutation approach'[2]. We engineered a series of cysteine variants of residues involved in CODD binding[2] using electrospray ionization MS (ESI-MS) to assay for successful enzyme-substrate cross-linking (**a**). The V314C/R281C and P317C/R281C PHD2 variants gave the best cross-linking yields with HIF1αNODD$_{395\text{-}413}$ (L397C/D412C) (NODD$_{DC}$). NODD$_{DC}$ was crystallized with the PHD2 C201A/R281C/P317C/R398A (**b**) or C201A/R281C/V314C/R398A (**c**) variant (PHD2$_{QM1}$ and PHD2$_{QM2}$, respectively) in complex with NOG and Mn(II). Substitution of C201A was carried out to avoid 'unwanted' disulfide formation with NODD$_{DC}$ involving the nucleophilic Cys201[7]. Structural analyses reveal that PHD2 and NODD$_{DC}$ (aa 400-413 including target P402, red arrow, **f**) adopt similar conformations in both PHD2$_{QM1}$.NODD$_{DC}$ and PHD2$_{QM2}$.NODD$_{DC}$ complexes (**e** and **f**). However, there are significantly larger differences in the NODD$_{DC}$ *N*-terminal region (aa 395-399, **f**), which includes a cross-linking site (black arrow). Crystal packing analyses (not shown) reveal that the β2/β3 loop and NODD are 2-fold non-crystallographic symmetry-related in the PHD2$_{QM2}$.NODD$_{DC}$ complex indicating a possible effect of crystal packing on NODD binding in this complex. We therefore, focused our work on the PHD2$_{QM1}$.NODD$_{DC}$ complex. Molecular dynamics (MD) simulations predict that, in solution, the overall structure of the PHD2.Fe(II).2OG.NODD$_{395\text{-}413}$ complex is very similar to that observed for the analogous PHD2$_{QM1}$.Mn(II).NOG.NODD$_{DC}$ crystal structure (Supplementary Fig. 3).

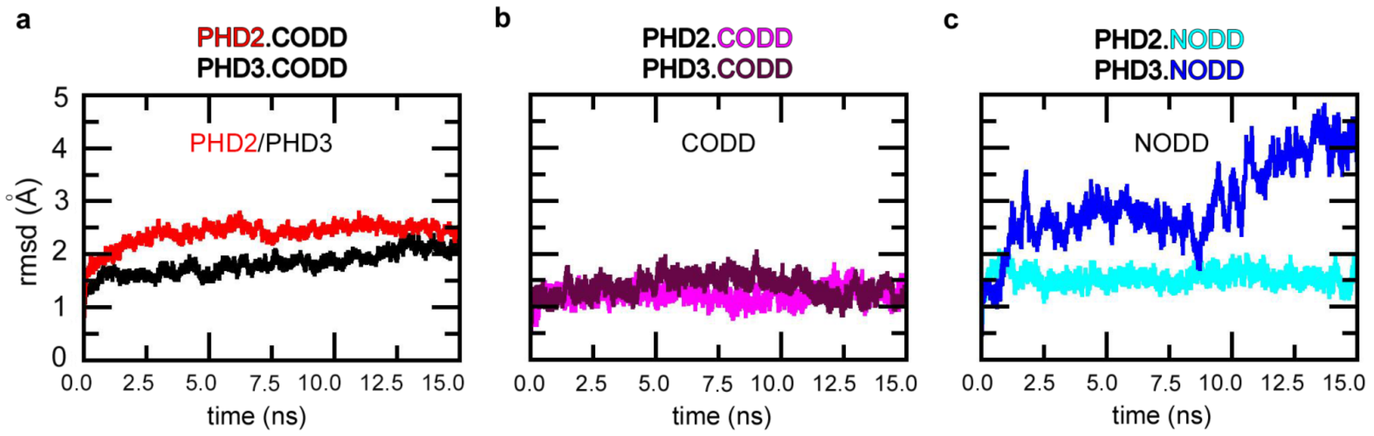

Supplementary Figure 3 | MD analyses on PHD2.CODD/NODD and PHD3.CODD/NODD modeled complexes. The MD simulations were carried out on models for PHD2.Fe(II).2OG.CODD (PHD2.CODD), PHD3.Fe(II).2OG.CODD (PHD3.CODD), PHD2.Fe(II).2OG.NODD (PHD2.NODD) and PHD3.Fe(II).2OG.NODD (PHD3.NODD) that were generated using crystal structures of PHD2.Mn.2OG.HIF-1αCODD$_{556\text{-}574}$ (PDB: 5L9B) and PHD2$_{QM1}$.Mn.NOG.HIF-1αNODD$_{DC(395\text{-}413)}$ (PDB: 5L9V) as templates. The panels show the Cα rmsd from the initial model as a function of simulation time for the complexes as indicated in figure titles by differently colored labels. The results of MD analyses on PHD2.CODD and PHD3.CODD modeled complexes suggest that PHD2 and PHD3 adopt similar folds in solution (**a**), and that the CODD conformations, and hence their binding modes, are similar when complexed with PHD2 or PHD3 (**b**). In contrast to the PHD2.CODD, PHD2.NODD and PHD3.CODD complexes, and consistent with the biochemical and cell based selectivity assay data[8-10], the PHD3.NODD complex is predicted to be substantially less stable by MD analyses (**c**).

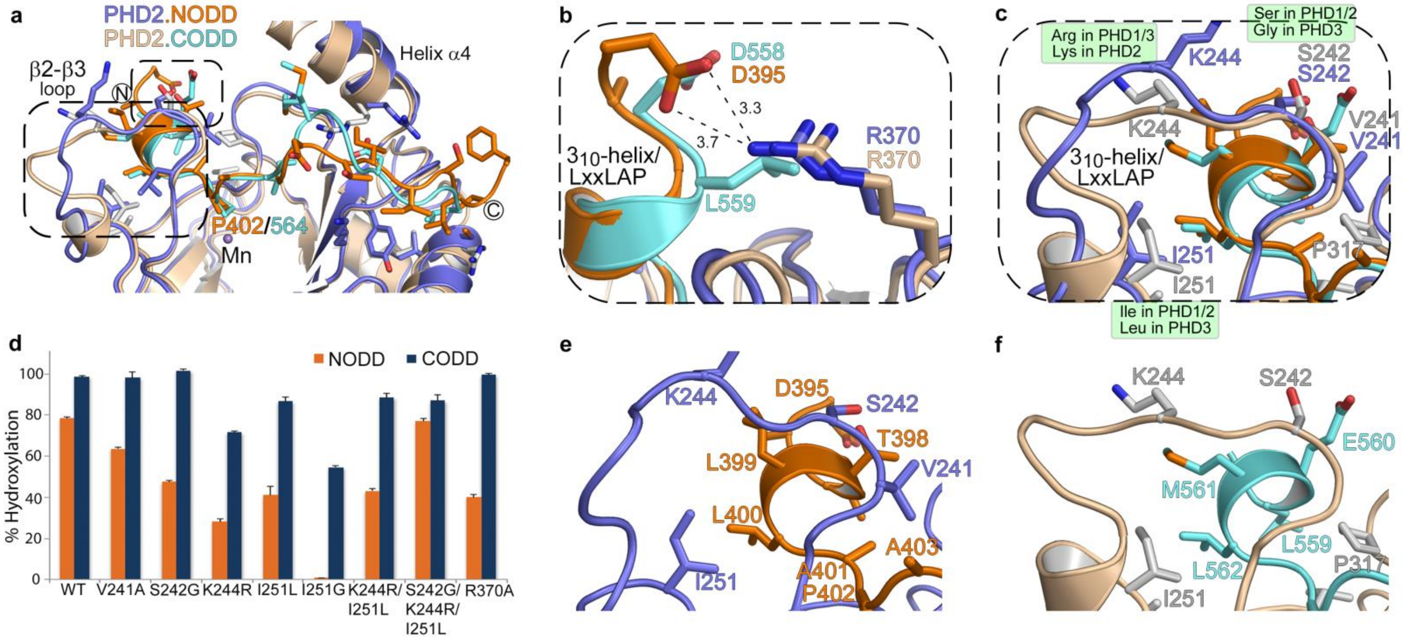

Supplementary Figure 4 | Binding of NODD/CODD residues *N*-terminal to the target prolines. (**a**)-(**c**) are views from the PHD2.NODD/CODD complex structures showing the multiple interactions in the *N*-terminal regions of NODD/CODD substrates with PHD2. (**a**) In the PHD2-substrate complexes, binding of NODD/CODD residues *N*-terminal to their target prolines involve PHD2 residues from βII (Tyr310, Val311, His313, Val314 and Asp315), a loop following βII (Pro317), the β2/β3 loop (Val241, Ser242, Lys244 and Ile251), βVIII (Trp389) and βIII (Arg322). (**b**) Compared to PHD2.CODD, the PHD2.NODD structure reveals that Asp395$_{NODD}$ forms a hydrogen-bond with Arg370$_{PHD2}$ (Asp395$_{NODD}$ Oδ1-NH1 Arg370$_{PHD2}$, 3.3 Å), which is located on the loop connecting PHD2 strands βVI-VII; this interaction was not observed in the PHD2.CODD complex although Arg370$_{PHD2}$ is positioned to make hydrophobic contacts with Leu559$_{CODD}$ (Leu559 Cδ1-Arg370 Cζ 3.3 Å) in the PHD2.CODD complex. (**c**), (**e**) and (**f**) highlight β2/β3 loop residues that interact with the substrate 3_{10}-helix/LXXLAP[11] motif. Notably, PHD2 residues Ser242, Lys244, Ile251 (non-conserved in PHDs) and Pro317 (a clinical variant site[12] that is conserved in PHD 1-3) are involved in determining substrate selectivity. (**d**) Endpoint assay results of NODD/CODD hydroxylations by PHD2 variants using MALDI-MS at an enzyme-substrate ratio (E:S) of 1:25. Data are mean and s.e.m. (n≥3).

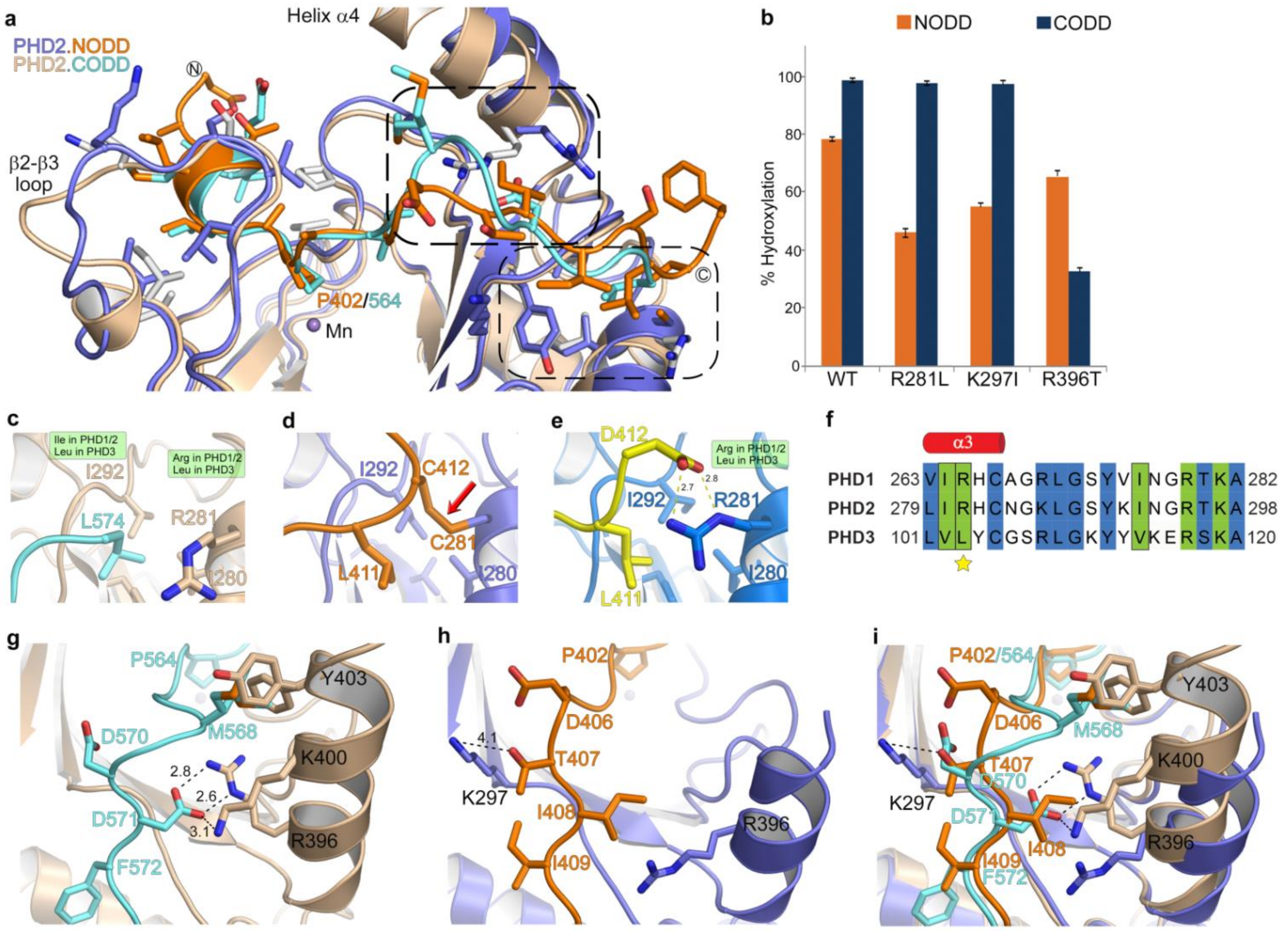

Supplementary Figure 5 | Binding of NODD/CODD residues *C*-terminal to the target proline(s). (**a**) Binding of ODD residues *C*-terminal to the target prolines involves PHD2 residues from βVIII (Trp389, Tyr390, Phe391), βIII (Arg322), helix α3 (Ile280, Arg281), a loop after α3 (Ile292, Asn293, Gly294, Arg295 and Lys297) and helix α4 (Arg396, Lys400 and Tyr403). (**b**) Endpoint assay results of NODD/CODD hydroxylations by the PHD2 variants using MALDI-MS at an E:S ratio of 1:25. Data are mean and s.e.m. (n≥3). Note, with respect to *C*-terminal substrate binding sites, we focused on helix α3 and the loop connecting α3 to βI, because all the other residues (on βIII, βVIII and α4) are conserved in PHD 1-3 (Supplementary Fig. 1). (**c**)-(**e**) *Comparison of the NODD C-terminal Asp412$_{NODD}$ binding sites in PHD2 and 3. Consistent with the PHD2.NODD structure, MD predict that Arg281$_{PHD2}$/Arg263$_{PHD1}$ (helix α3) hydrogen bonds with Asp412$_{NODD}$ in solution* (**e**)*; PHD3 possesses a leucine residue (Leu103$_{PHD3}$) at this site* (**f**)*, so disfavors binding of the acidic Asp412$_{NODD}$ sidechain*. Substitution of Arg281$_{PHD2}$ with a leucine leads to little or no reduction in activity with CODD, but a significant reduction (>40%) in activity with NODD (**b**). This effect is more pronounced in a NODD/CODD competition experiment, where NODD was hydroxylated to about <10% when using a 1:25 E/S ratio and remained almost completely unmodified using a 1:50 E/S ratio (Supplementary Table 2). Compared to NODD, CODD makes additional interactions with the *C*-terminal helix α4 (Arg396, Lys400 and Tyr403) including a salt-bridge between Asp571$_{CODD}$-Arg396$_{PHD2}$ that are absent in the PHD2.NODD complex (**g**-**i**); as a result, PHD2 α4 helix in the PHD2.CODD complex moves towards CODD/the active site compared to PHD2.NODD or the substrate-unbound PHD2.2OG complex. NODD has more hydrophobic residues than CODD in its *C*-terminal region (in particular the three sequential aspartyl residues in CODD are substituted in NODD by ^{407}Thr-Ile-Ile^{409}) and employs hydrophobic interactions for binding (**h**-**i**).

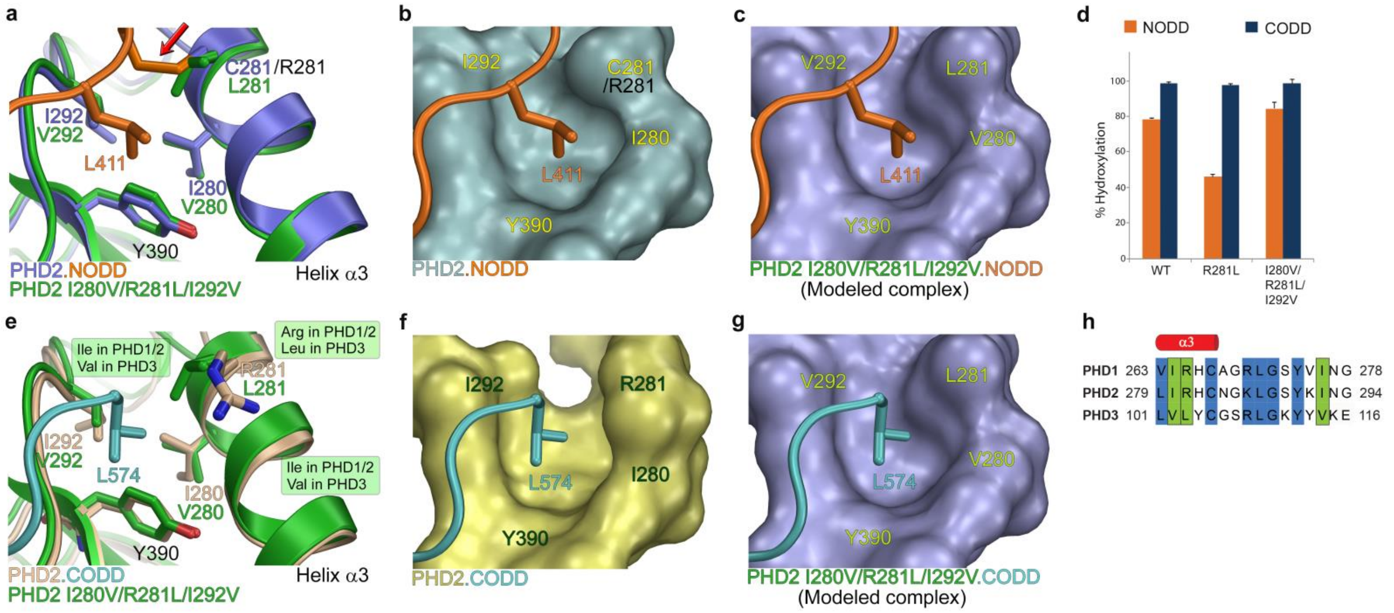

Supplementary Figure 6 | The roles of *C*-terminal Leu411$_{\mathrm{NODD}}$/Leu574$_{\mathrm{CODD}}$ binding site residues (Ile280$_{\mathrm{PHD2}}$ /helix α3, Ile292$_{\mathrm{PHD2}}$ /α3-βI loop and Tyr390$_{\mathrm{PHD2}}$/βVIII) in determining NODD/CODD selectivity. Leu574$_{\mathrm{CODD}}$ is important for HIF-1α hydroxylation in cells[13]. The predicted Leu574$_{\mathrm{CODD}}$ binding site in PHD3 is different compared to those of PHD1/2 in terms of the size of the cleft as it has two valines (Val102$_{\mathrm{PHD3}}$, and Val114$_{\mathrm{PHD3}}$) compared to two isoleucines in PHD1/2 (**h**). A I280V/I292V/R281L triple variant hydroxylates both ODDs (NODD to higher levels than PHD2 R281L) (**d**). Data are mean and s.e.m. (n≥3). Consistent with the endpoint assay results, kinetics show that, although $k_{\mathrm{cat(CODD)}}$ remained unchanged, K_{m} reduced almost half to that observed for the R281L variant (Supplementary Table 3); both K_{m} and k_{cat} for NODD slightly improved (compared to the R281L). To investigate the structural basis of catalytic differences between the wt, R281L and I280V/I292V/R281L PHD2 variants, the triple variant was crystallized in the $P6_3$ form; the structure reveals that the multiple substitutions create a wider hydrophobic pocket (compare **b** and **c**; **f** and **g**) at this site that likely accommodates the leucine more efficiently. Support for this proposal comes from yeast-two hybrid experiments where except for phenylalanine, substitution of Leu574$_{\mathrm{CODD}}$ with all other residues is disfavored for a stable interaction with PHD3; PHD2 has a slightly higher preference for valine (shorter) over leucine at this position[14].

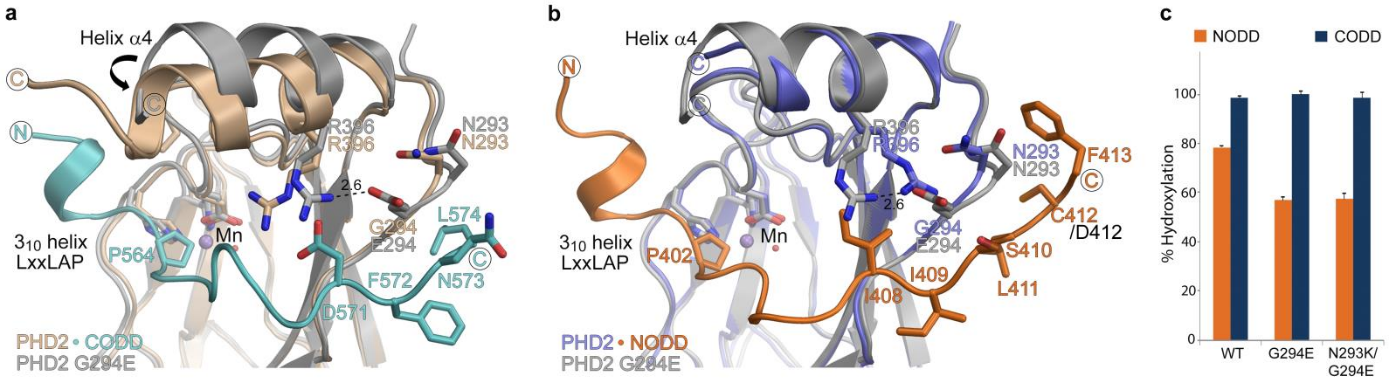

Supplementary Figure 7 | Views from superimposed structures of the G294E variant (grey) with PHD2.CODD (a) and PHD2.NODD (b). Modeling studies indicated that the Glu116$_{PHD3}$ (analogous to Gly278$_{PHD1}$/Gly294$_{PHD2}$) side-chain in PHD3 might alter substrate binding when compared to PHD2.CODD/NODD complexes. We tested the effects of Asn293$_{PHD2}$ and Gly294$_{PHD2}$, which are located in the *C*-terminal substrate binding region and are replaced in PHD3 by Lys115$_{PHD3}$ and Glu116$_{PHD3}$ on PHD catalysis. The PHD2 G294E and N293K/G294E variants showed 20-25% reductions in NODD, but less significant/unaltered levels of CODD hydroxylations (**c**). Data are mean and s.e.m. ($n \geq 3$). A G294E variant crystal structure reveals local changes around Glu294 (in the variant) especially involving the sidechain of Arg396$_{PHD2}$ (**a** and **b**). In the PHD2.CODD structure (**a**), Arg396$_{PHD2}$ moves towards CODD to form a salt-bridge with Asp571$_{CODD}$ compared to PHD2.NODD complex (**b**), where the residue adopts a conformation that would cause a steric clash with Glu294. In the G294E variant structure, Arg396$_{PHD2}$ takes a different conformation similar to that observed in the PHD2.Mn.NOG or the PHD2.Fe.2OG complexes[15], in which it hydrogen bonds with Glu294. Therefore, although Gly294 to glutamate substitution may not directly affect substrate binding, a glutamate at this position likely modifies the Arg396$_{PHD2}$ sidechain orientation. Note that the PHD2 G294S mutation present in a cervix carcinoma clinical sample[4] may show similar differences in reactivity.

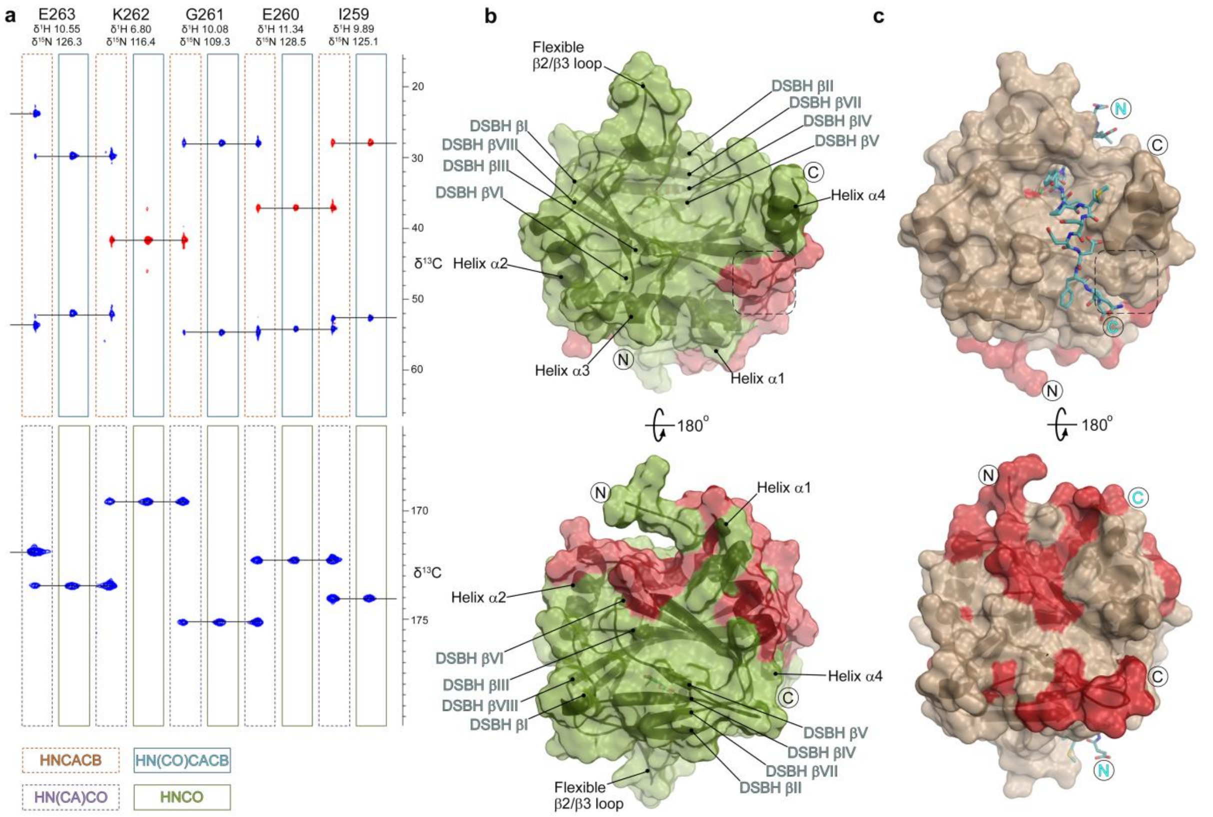

Supplementary Figure 8 | Backbone assignment of PHD2$_{181\text{-}402}$. (**a**) Example stripes extracted from the ^{1}H-^{15}N planes of four 3D triple-resonance experiments (HNCACB, HN(CO)CACB, HN(CA)CO and HNCO) illustrating the sequential assignment of residues Ile259 to Glu263 linked by the horizontal crosspeak correlations. Chemical shifts are deposited in the Biological Magnetic Resonance Data Bank (BMRB) with deposition codes, 26741 and 26742 for ^{2}H,^{13}C,^{15}N-PHD2$_{181\text{-}402}$.Zn(II).2OG and ^{2}H,^{13}C,^{15}N-PHD2$_{181\text{-}402}$.Zn(II).2OG.CODD, respectively. Following the complete assignment process, a significant number of residues were assigned as having multiple resonances (marked red in **b** and **c**). These peaks (mostly duplicate) often have different ^{1}H-^{15}N shifts but almost identical ^{13}C shifts for Cα and Cβ. Multiple peaks for the same residues imply different local conformations that are in slow exchange; note, most of the multiple peaks have significantly lower intensities (usually <10%) than the corresponding major peaks. ZZ-exchange experiments were attempted to investigate the exchange rates between these duplicate peaks; no cross peaks were observed (with mixing time of 200 ms and a relaxation delay of 3 seconds), indicating these conformers are in slow exchange with each other and that the exchange rate is slower than the NMR time scale. (**b**) Residues assigned as having multiple peaks concentrate at the *N*-terminal region of PHD2 including the *N*-terminus (aa 181-184), α1 (aa 189-195 and aa 200-204), the regions between β1 and α2 (aa 210-219), between α3 and β4 (aa 283-294) and at the base of helix α4 (aa 393-395). (**c**) On CODD binding, the residues with multiple peaks concentrate in: the *N*-terminus (aa 181-186; helix α1, aa 189-193), the regions between β1 and α2 (aa 208-219) and between α3 and β4 (aa 284-288) and the DSBH core (βI, aa 299-302; βIII, aa 329-331; βIV, aa 340-345; βIV-V loop, aa 349-354). Note, CODD binding reduces the slow-exchange behaviors for the regions between α3 and β4 (aa 283-294) and the base of helix α4 (aa 393-395) (shown boxed in black in **b** and **c**).

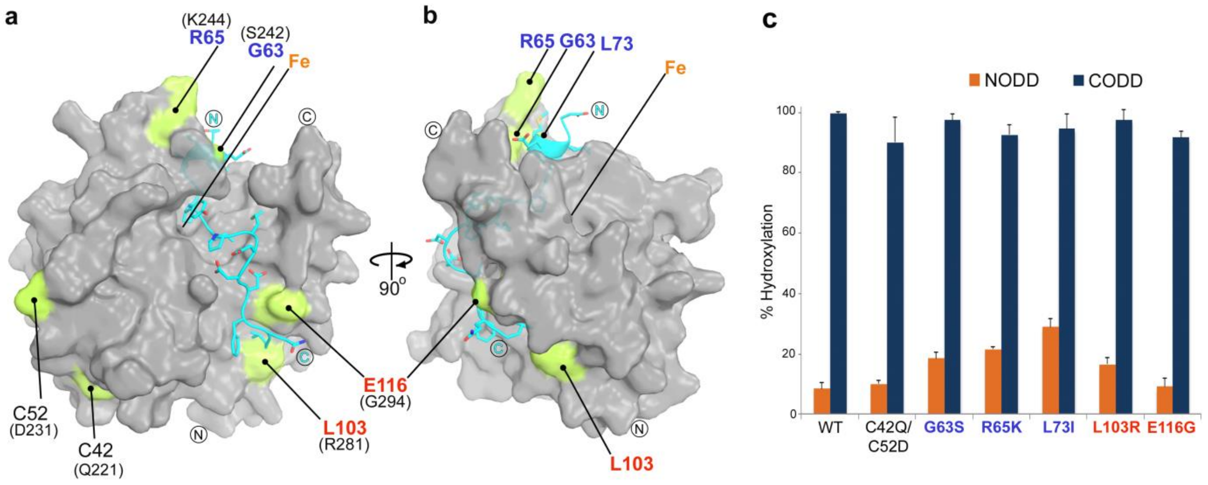

Supplementary Figure 9 | Combined modelling and biochemical analyses identify ODD selectivity determinants in PHD3. Figures (**a**) and (**b**) show surface representations of a PHD3.CODD complex that was modelled based on the PHD2.CODD crystal structure (PDB: 5L9B). MD predict that the overall binding of CODD to both PHD2 and PHD3 (and hence CODD conformations in both complexes) is similar (see Supplementary Fig. 3). We produced a series of PHD3 point variants with the aim of making PHD3 more 'PHD2 like' to increase its ability to hydroxylate NODD. Of these variants, R65K and L73I PHD3 clearly increased NODD hydroxylation relative to wt PHD3 (25-30% as compared to <10%). A recent study suggests that PHD3 forms oligomers in the presence of metals such as Zn(II), via reaction of two surface cysteines Cys42 and Cys52 that are replaced by Gln221 and Asp231 in PHD2[16]. We produced a double cysteine variant of PHD3 (C42Q/C52D) that manifests improved behavior during purification and has same activity as wt PHD3 (**c**). Data are mean and s.e.m. (n≥3).

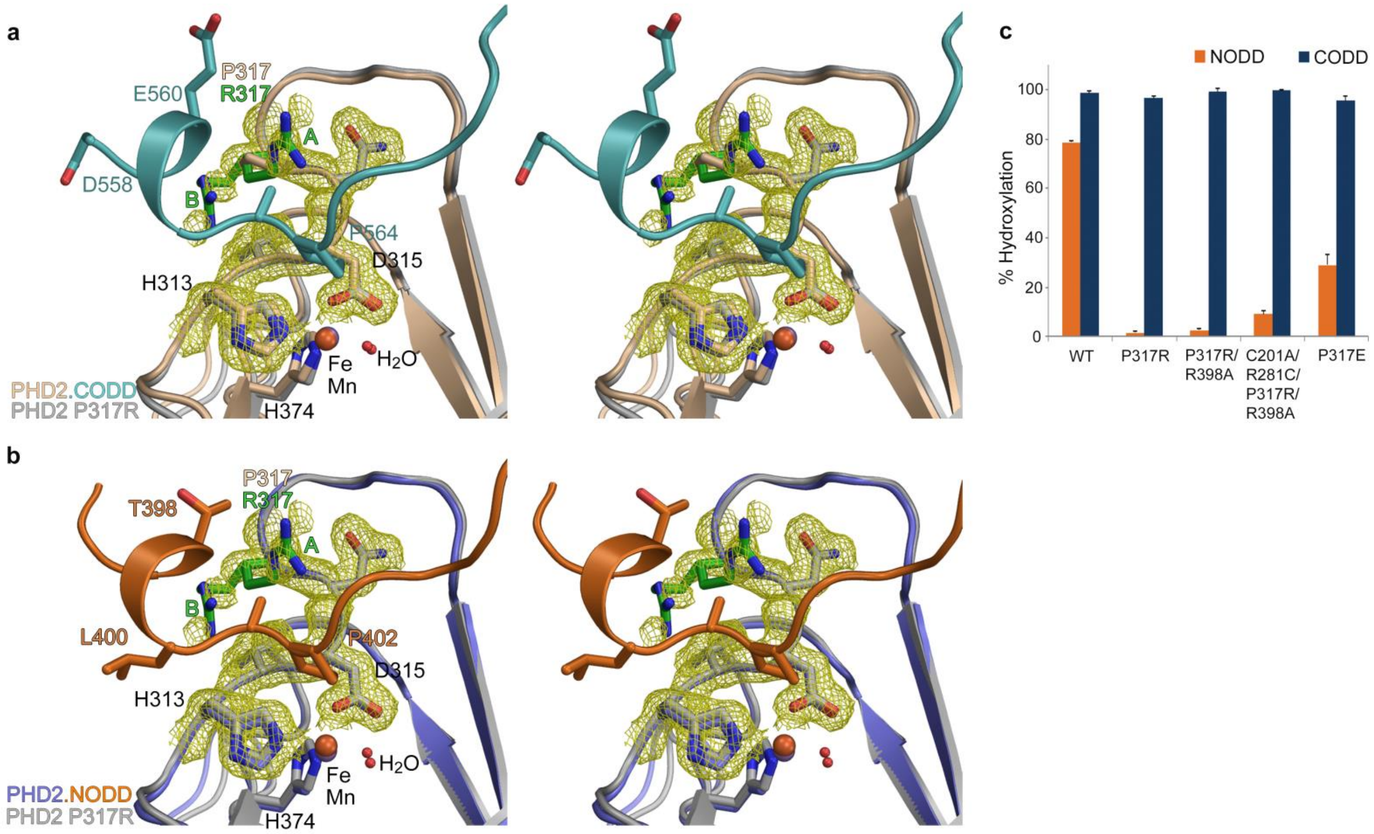

Supplementary Figure 10 | Stereoviews from superimposed crystal structures of PHD2 P317R (grey) and PHD2.CODD (a) and PHD2.NODD (b). Electron density map (simulated annealed $2F_o$-F_c contoured to 1σ) for residues Asp313-Arg317 in the PHD2 P317R crystal structure. *P317R PHD2 is the most frequently occurring PHD2 mutation that has been associated with familial erythrocytosis*[12,17]. P317R PHD2 (full-length) has been reported to cause a reduction in HIF-1$\alpha_{549\text{-}575}$ hydroxylation (<10% of wt PHD2) in a VHL-capture assay[12]. *The PHD2 P317R variant retains (almost) full activity with CODD compared to wt PHD2* (see also Fig. 2b for kinetic data, k_{cat}/K_m 42 × 10^2 M^{-1}s^{-1} compared to 46 × 10^2 M^{-1}s^{-1} for wt PHD2*), but the variant strikingly does not hydroxylate NODD* (**c**). Data are mean and s.e.m. (n≥3). A crystal structure of PHD2 P317R reveals a similar overall fold to PHD2; Arg317$_{PHD2}$ (highlighted in green) was in 2 conformations with equal occupancy. In one of these conformations, the Arg317$_{PHD2}$ sidechain likely enables the P317R variant to interact with the LXXLAP/3$_{10}$ helix. However, the other Arg317$_{PHD2}$ conformation appears to clash with Leu559$_{CODD}$/Leu397$_{NODD}$ of the LXXLAP motif.

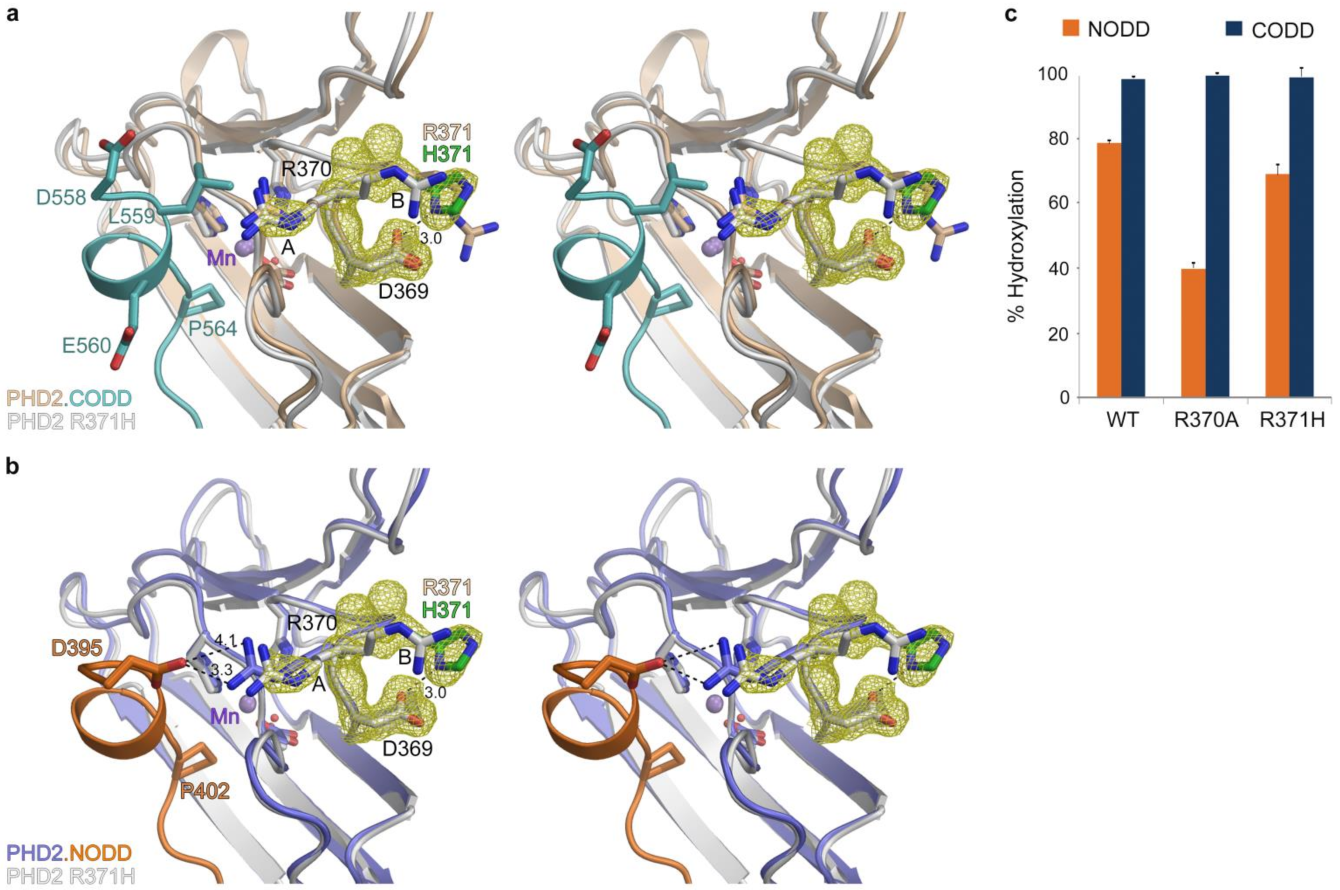

Supplementary Figure 11 | Stereoviews from superimposed crystal structures of PHD2 R371H (grey) and PHD2.CODD (a) and PHD2.NODD (b). Electron density map (simulated annealed 2F_o-F_c contoured to 1σ) for residues Asp369-His371 in the PHD2 R371H crystal structure. Heterozygous mutations of PHD2 R371H occur in patients with familial erythrocytosis[18]. Arg371$_{PHD2}$ is conserved in all human PHDs and forms an internal salt-bridge interactions with Asp369$_{PHD2}$ (Asp369 Oδ1-Arg371 Nε 3.2 Å; Asp369 Oδ2-Arg371 NH2 3.0 Å). PHD2 R371H retains >60% activity with NODD, but is equally active as wildtype with CODD (**c**). Data are mean and s.e.m. (n≥3). Kinetics show that the R371H variant hydroxylates both substrates with much lower efficiency relative to wt PHD2 (k_{cat}/K_m reduces to almost half for both NODD and CODD, Fig. 2b). Given that the K_m (CODD) remained almost unchanged while that for NODD reduced (Supplementary Table 3), the loss of catalytic efficiency could be due to a reduction in the reaction rate of the enzyme-substrate complex (as reflected by significantly low k_{cat} for both ODDs) as well as substrate binding (i.e. K_m). A crystal structure of the R371H variant reveals that substitution of Arg371$_{PHD2}$ by a histidine (highlighted in green) causes (partial) loss of the electrostatic interaction with Asp369$_{PHD2}$ which in turn leads to repositioning of the Arg370$_{PHD2}$ sidechain (that interacts with Asp395$_{NODD}$); the conformational change in Arg370$_{PHD2}$ sidechain likely contributes to reduced NODD hydroxylation by the R371H variant.

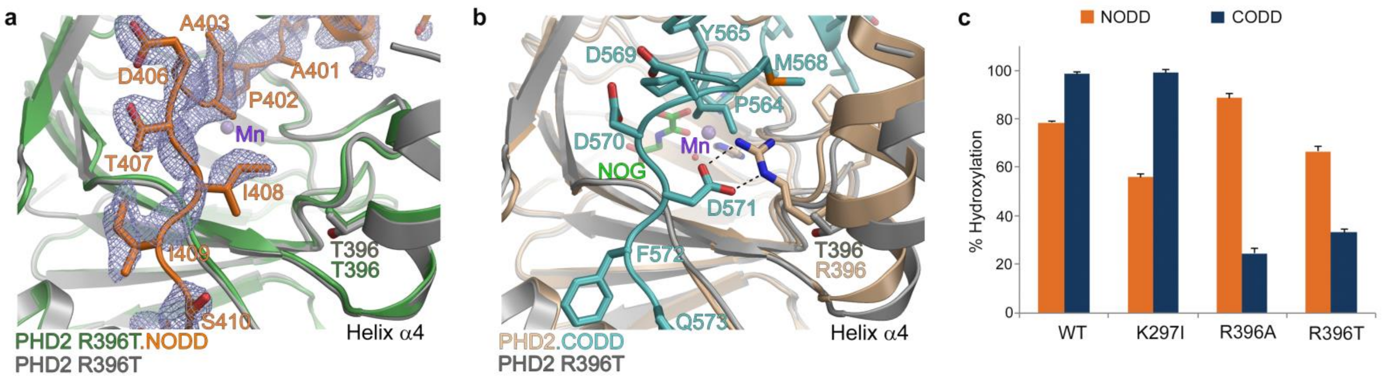

Supplementary Figure 12 | Views from crystal structures of PHD2 R396T (with and without NODD, orange) showing the F_o-F_c OMIT map contoured to 3σ around NODD. The PHD2 R396T variant present in breast carcinoma[4] and R396A were similarly efficient at hydroxylating NODD as PHD2 wt; however, there was a marked loss of CODD activity as characterized by endpoint assays (**c**), slow initial rates and high K_m values (> 300 μM) (Supplementary Table 3). Data are mean and s.e.m. (n≥3). (**a**) Overlays of crystal structures of the R396T variant alone or in complex with NODD reveal threonine substitution at this position would not directly interfere with NODD binding. (**b**) Superimposition of PHD2 R396T variant and PHD2.CODD crystal structures reveal loss of electrostatic interaction of the R396T variant with $Asp571_{CODD}$.

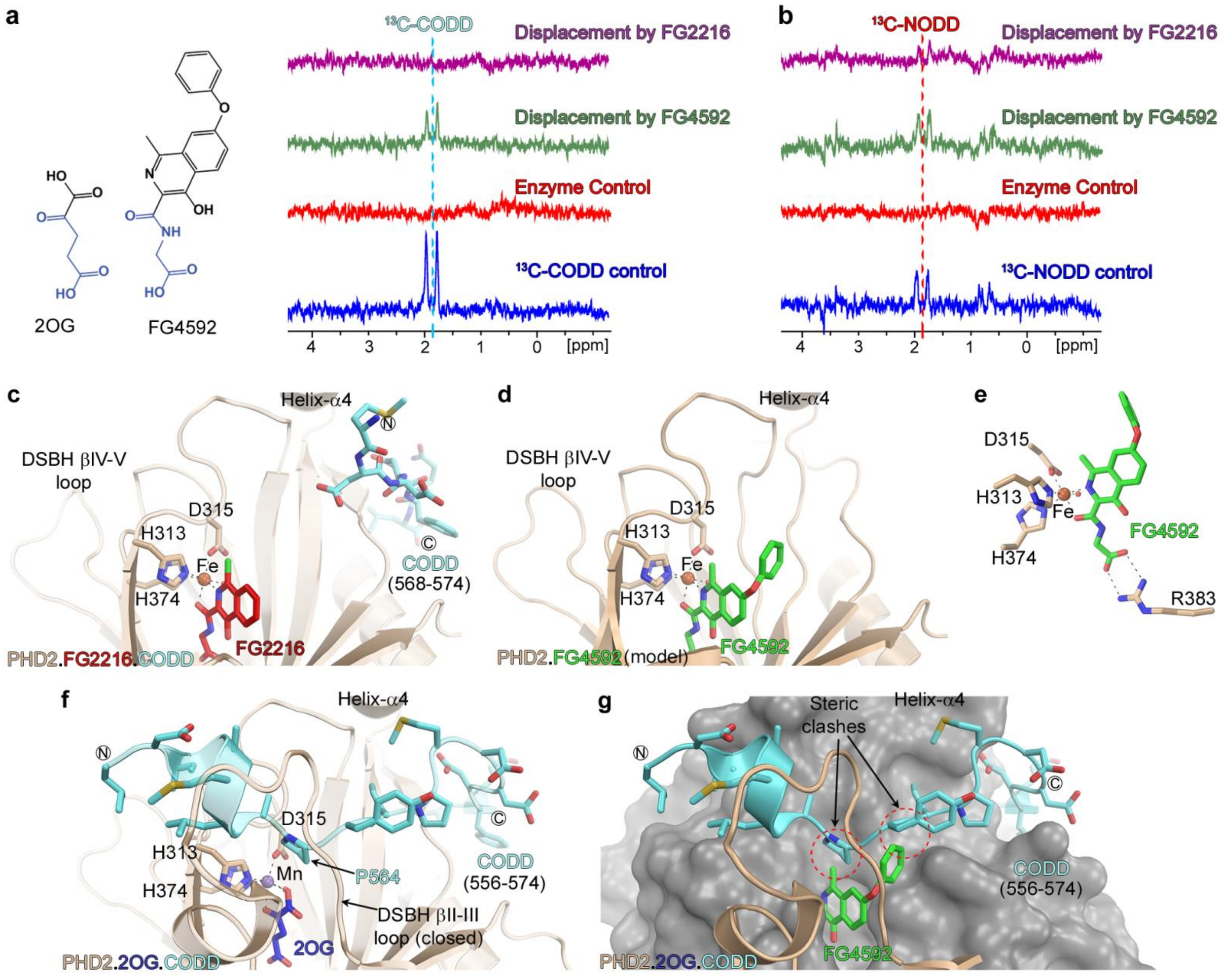

Supplementary Figure 13 | Views from PHD2.Fe.FG2216.CODD (PDB: 3HQU) and PHD2.Mn.2OG.CODD (PDB: 5L9B) crystal structures and a PHD2.Fe.FG4592 model indicating the extent to which the PHD inhibitors, FG2216 and FG4592 (Roxadustat) are predicted to differentially displace CODD and NODD. FG2216 and FG4592 both manifest efficient NODD displacement from PHD2 whereas effective CODD displacement only occurs with FG4592 as evidenced by 1D ^{13}C-selective Clean In-Phase (CLIP) HSQC NMR (**a** and **b**). See Methods for detail. (**c**) A crystal structure of the PHD2.FG2216.CODD complex (PDB: 3HQU) coupled to mass spectrometric studies (not shown) reveals that PHD2 can form a ternary complex with CODD in the presence of FG2216 wherein CODD employs residues C-terminal to target proline (P564) for binding PHD2. In this crystal form ($P6_3$), the CODD residues N-terminal to P564, including the conserved LXXLAP domain, were disordered likely due to crystal packing issues[2]. (**d**) and (**e**) show views from a PHD2.Fe.FG4592 model that was generated by using a structure for PHD2.Mn.FG2216 (PDB: 4BQX) and a close-up view from the PHD2.Fe.FG4592 model active site. (**f**) and (**g**) show views from a PHD2.Mn.2OG.CODD complex structure (PDB: 5L9B) and a superimposed view of the PHD2.Fe.FG4592 model and the PHD2.Mn.2OG.CODD complex structure indicating how the phenoxy group in FG4592 (which is not present in FG2216) will collide with ODD binding.

Supplementary Tables

Supplementary Table 1 | Summary of results for 2OG turnover and hydroxylation assays with NODD and CODD with PHD2 and PHD2 variants (using an enzyme-substrate ratio of 1:25). In the mechanism of 2OG-oxygenase-catalyzed reactions[19,20], one substrate molecule is normally hydroxylated per molecule of 2OG-decarboxylated. However, this 'coupling ratio' can be less than unity ('substrate uncoupled turnover') as reported for some PHD2 variants, e.g. where the active site is exposed as a consequence of deleting the β2/β3 loop residues that isolate the active site or where the β2/β3 loop position is forced to a relatively 'open' conformation[2,9]. The coupling ratios for the selected variants were determined by ^{14}C-labelled 2OG-decarboxylation assays of PHD reactions[2,9].

Wildtype/ Variant PHD2	Equivalent residues in PHD1-3 isoforms			% 2OG turned over relative to PHD2*		% Hydroxylation	
	PHD1	PHD2	PHD3	CODD	NODD	CODD	NODD
PHD2	-	-	-	100.0	100.0	98.5 ± 0.7	78.5 ± 0.7
R281L	Arg 265	Arg 281	Leu 103	92.2	51.7	97.5 ± 0.7	46.0 ± 1.4
P317R	Pro 301	Pro 317	Pro 139	96.1	20.2	96.5 ± 0.7	n.d.
R371H	Arg 355	Arg 371	Arg 193	106.6	89.8	99.0 ± 2.8	69.0 ± 2.8
R396A	Arg 380	Arg 396	Arg 218	29.3	90.1	28.5 ± 5.0	96.5 ± 0.7
K244R/I251L	Arg 228<br>Ile 235	Lys 244<br>Ile 251	Arg 65<br>Leu 73	112.0	91.2	88.5 ± 2.1	43.0 ± 1.4
S242G/K244R/I251L	Ser 226<br>Arg 228<br>Ile 235	Ser 242<br>Lys 244<br>Ile 251	Gly 63<br>Arg 65<br>Leu 73	104.8	103.5	97.0 ± 2.8	77.0 ± 1.4
I280V/I292V/R281L	Ile 264<br>Arg 265<br>Ile 276	Ile 280<br>Arg 281<br>Ile 292	Val 102<br>Leu 103<br>Val 114	112.3	60.2	98.5 ± 2.1	84.5 ± 3.5

* Activities measured in nmoles of 2OG turnover (mean ± s.e.m.) were converted into percentages relative to PHD2 and hence standard deviations are not given.
n.d. = not detected under the experimental conditions; N.T. = Not tested.
NODD = HIF-1$\alpha_{395-413}$; CODD = HIF-1$\alpha_{556-574}$.

Supplementary Table 2 | Summary of results for % hydroxylation of HIF-1α ODDs (NODD and CODD) at variable E:S ratios (1:50 and 1:25) using wt PHD2 or the important PHD2 variants in individual and competition substrate (1:1) experiments. NODD/CODD hydroxylation was measured by MALDI-TOF mass spectrometry with appropriate controls[2]. Data are mean and s.e.m. (n≥3).

PHD2wt/varaints	CODD (HIF-1$\alpha_{556-574}$)				NODD (HIF-1$\alpha_{395-413}$)			
	Individual (1:50)	Individual (1:25)	Competition (1:50)	Competition (1:25)	Individual (1:50)	Individual (1:25)	Competition (1:50)	Competition (1:25)
PHD2	97.5 ± 0.7	98.5 ± 0.7	99.5 ± 0.7	99.5 ± 0.7	76.0 ± 1.4	78.5 ± 0.7	9.5 ± 0.7	50.0 ± 2.8
S242G	96.5 ± 0.7	97.5 ± 0.7	N.T.	98.5 ± 0.7	33.5 ± 0.7	47.5 ± 0.7	N.T.	24.0 ± 0.7
K244R	64.0 ± 1.4	71.5 ± 0.7	N.T.	63.5 ± 3.5	22.0 ± 2.8	28.0 ± 1.4	N.T.	n.d.
I251G	36.0 ± 2.8	54.5 ± 0.7	N.T.	46.0 ± 2.8	10.0 ± 0.7	15.0 ± 2.8	N.T.	n.d.
I251L	69.0 ± 1.4	86.5 ± 0.7	N.T.	N.T.	28.0 ± 0.7	41.0 ± 0.7	N.T.	N.T.
R281L	89.0 ± 1.4	97.5 ± 0.7	97.0 ± 1.4	100.0 ± 0	29.0 ± 0.0	46.0 ± 1.4	n.d.	9.5 ± 0.7
G294E	41.5 ± 0.7	62.5 ± 2.1	N.T.	52.0 ± 1.4	<10	34.5 ± 0.7	N.T.	n.d.
P317R	93.0 ± 1.4	96.5 ± 0.7	96.5 ± 0.7	97.5 ± 0.7	n.d.	n.d.	n.d.	n.d.
P317E	90.5 ± 2.1	95.5 ± 2.1	97.5 ± 2.1	96.0 ± 1.4	11.0 ± 1.4	29.0 ± 4.2	n.d.	<10
R371H	98.5 ± 3.5	99.0 ± 2.8	95.5 ± 0.7	97.5 ± 2.1	55.0 ± 1.4	69.0 ± 2.8	n.d.	35.5 ± 2.1
R396A	29.0 ± 4.2	28.5 ± 5.0	11.5 ± 0.7	24.5 ± 2.1	74.0 ± 2.8	96.5 ± 0.7	81.5 ± 0.7	88.5 ± 2.1
K244R/I251L	86.5 ± 0.7	88.5 ± 2.1	96.5 ± 0.7	98.5 ± 0.7	43.0 ± 1.4	43.0 ± 1.4	n.d.	39.5 ± 0.7
S242G/K244R/I251L	90.5 ± 2.1	97.0 ± 2.8	97.5 ± 2.1	99.0 ± 1.4	69.0 ± 1.4	77.0 ± 1.4	n.d.	n.d.
I280V/R281L/I292V	90.5 ± 2.1	98.5 ± 2.1	93.5 ± 0.7	96.5 ± 6.4	69.0 ± 1.4	76.5 ± 0.7	19 ± 2.8	32.0 ± 2.8

n.d. = not detected under the experimental conditions; N.T. = Not tested.
NODD = HIF-1$\alpha_{395-413}$; CODD = HIF-1$\alpha_{556-574}$.

Supplementary Table 3 | Kinetic analyses of the PHD2 variants for the hydroxylation of CODD (HIF-1$\alpha_{556-574}$) and NODD (HIF-1$\alpha_{395-413}$) substrates employing a MALDI-MS based assay (n = 3-9).

**PHD2 wt/ variants**	**CODD** (HIF-1$\alpha_{556-574}$)			**NODD** (HIF-1$\alpha_{395-413}$)		
	k_{cat} (Mean ± SEM) s^{-1}	K_m (Mean ± SEM) µM	k_{cat}/K_m $M^{-1}s^{-1}$ ×10^2	k_{cat} (Mean ± SEM) s^{-1}	K_m (Mean ± SEM) µM	k_{cat}/K_m $M^{-1}s^{-1}$ ×10^2
WT	0.0603 ± 0.0010	13.08 ± 1.12	46.1	0.0480 ± 0.0015	35.26 ± 2.55	13.6
S242G	0.0252 ± 0.0005	5.96 ± 0.56	42.3	0.0133 ± 0.0002	17.43 ± 0.83	7.6
K244R	0.0334 ± 0.0012	16.59 ± 1.70	20.1	0.0109 ± 0.0002	25.21 ± 0.92	4.3
I251G	0.0321 ± 0.0090	>100	-	n.d.	n.d.	-
I251L	0.0707 ± 0.0016	24.79 ± 1.46	28.5	0.0121 ± 0.0003	69.78 ± 3.23	1.7
R281L	0.0352 ± 0.0016	19.88 ± 2.41	17.7	0.0192 ± 0.0007	113.2 ± 6.04	1.7
P317R	0.0302 ± 0.0004	7.23 ± 0.48	41.8	n.d.	n.d.	-
R371H	0.0279 ± 0.0006	13.70 ± 1.03	20.3	0.0159 ± 0.0003	26.45 ± 1.08	6.0
R396A	-	>300	-	0.0582 ± 0.0013	52.91 ± 2.31	11.0
R396T	-	>300	-	0.0390 ± 0.0028	79.66 ± 9.74	4.9
K244R/I251L	0.0579 ± 0.0015	21.17 ± 1.97	27.4	0.0140 ± 0.0003	50.44 ± 1.90	2.8
S242G/K244R/I251L	0.0339 ± 0.0007	7.40 ± 0.99	45.8	0.0139 ± 0.0002	28.34 ± 1.03	4.9
I280V/R281L/I292V	0.0336 ± 0.0009	9.57 ± 1.37	35.1	0.0212 ± 0.0005	61.56 ± 2.82	3.4

n.d. = initial rates could not be determined due to the low level of hydroxylation (<10%) observed under the experimental conditions; k_{cat}/K_m values are calculated from the average k_{cat} and K_m values for wt and variant PHD2.

Supplementary Table 4 | NMR data collection conditions.

	**Triple resonance**	**^{1}H-^{15}N HSQC**	**^{15}N-relaxation (T_1/T_2) and ^{1}H-^{15}N NOE**	**CLIP-HSQC**
**Sample composition**				
PHD2.2OG	400 µM ^{2}H,^{13}C,^{15}N-labelled $PHD2_{181\text{-}402}$, 690 µM $ZnCl_2$, 920 µM 2OG and 67 µM DSS.	50-400 µM ^{2}H,^{13}C,^{15}N-labelled $PHD2_{181\text{-}402}$ (or ^{15}N-labelled $PHD2_{181\text{-}402}$), 75-690 µM $ZnCl_2$, 100-920 µM 2OG, 0-67 µM DSS	1 mM ^{15}N-$PHD2_{181\text{-}402}$, 1.5 mM $ZnCl_2$ and 2 mM 2OG.	50 µM $PHD2_{181\text{-}426}$, 400 µM $ZnCl_2$, and 50 µM 1,2,3,4-^{13}C-labelled 2OG
PHD2.CODD$^{\psi}$	400 µM ^{2}H,^{13}C,^{15}N-labelled $PHD2_{181\text{-}402}$, 690 µM $ZnCl_2$, 920 µM 2OG, 800 µM CODD and 67 µM DSS.	50-400 µM ^{2}H,^{13}C,^{15}N-labelled $PHD2_{181\text{-}402}$ (or ^{15}N-labelled $PHD2_{181\text{-}402}$), 75-690 µM $ZnCl_2$, 100-920 µM 2OG, 250-800 µM CODD, 0-67 µM DSS	400 µM ^{15}N-$PHD2_{181\text{-}402}$, 600 µM $ZnCl_2$, 800 µM 2OG and 800 µM CODD.	50 µM $PHD2_{181\text{-}426}$, 400 µM Zn(II), 50 µM 1,2,3,4-^{13}C-labelled 2OG and 50 µM CODD (U-^{13}C and U-^{15}N labelled on P564)
PHD2.NODD$^{\psi}$	n.d.	50 µM ^{15}N-$PHD2_{181\text{-}402}$, 75 µM $ZnCl_2$, 100 µM 2OG and 25.6–400 µM NODD.	n.d.	50 µM $PHD2_{181\text{-}426}$, 400 µM Zn(II), 50 µM 1,2,3,4-^{13}C-labelled 2OG and 50 µM NODD (U-^{13}C and U-^{15}N labelled on P402)
**Data collection**				
Spectrometer	Bruker Avance III (700 MHz) with cryogenic probe	Bruker Avance III (900 MHz) with cryogenic probe	Bruker Avance III (900 MHz) with cryogenic probe	Bruker Avance III (700 MHz) with cryogenic probe
Temp (°K)	310	310	310	298
NMR tube used	Shigemi tubes (3 mm, matched with D_2O)	Shigemi tubes (5 mm, matched with D_2O)	Shigemi tubes (5 mm, matched with D_2O)	Bruker MATCH tubes (3 mm)

All samples were buffered with 50 mM Tris-D11pH 6.6 and 0.02% NaN_3 in H_2O-D_2O (9:1).
DSS (4,4-dimethyl-4-silapentane-1-sulfonic acid) was used as a reference for chemical shifts.
n.d., not performed due to the limited solubility of NODD.
$^{\psi}$ Substrate peptides used, NODD: ^{395}DALTLLAPAAGDTIISLDF^{413};
CODD: ^{556}DLDLEMLAPYIPMDDDFQL^{574};
Data were processed using Bruker TopSpin 3.1, and analyzed using CcpNmr Analysis software [21].

Supplementary Table 5 | Buffer and vapor diffusion conditions used for crystallization.

Protein complex#	Sample composition$^{\psi}$	Crystallization conditions	Vapor diffusion conditions
PHD2•NOG	1mM PHD2 + 1.2mM $MnCl_2$ + 2mM NOG	0.1 M HEPES-Na pH 7.5, 2% v/v Polyethylene glycol 400, 2.0 M ammonium sulfate	Sitting drop (300 nl), protein-to-well ratio, 1:1, 293K
PHD2•2OG•CODD	1mM PHD2 + 1.2mM $MnCl_2$ + 2mM 2OG + 2mM CODD	0.2 M Magnesium chloride hexahydrate, 0.1 M BIS-TRIS pH 5.5, 25% w/v polyethylene glycol 3350	Sitting drop (300 nl), protein-to-well ratio, 2:1, 293K
PHD2-QM1•NOG•$NODD_{DC}$	1mM PHD2-QM1 + 1.2mM $MnCl_2$ + 2mM 2OG + 2mM $NODD_{DC}$	0.2 M ammonium chloride, 20 % w/v polyethylene glycol 3350	Sitting drop (300 nl), protein-to-well ratio, 1:2, 293K
PHD2-QM2•NOG•$NODD_{DC}$	1mM PHD2-QM2 + 1.2mM $MnCl_2$ + 2mM 2OG + 2mM $NODD_{DC}$	0.1 M HEPES pH 7.5, 2.0 M ammonium sulfate	Sitting drop (300 nl), protein-to-well ratio, 2:1, 293K
PHD2-QM1-R396T•NOG•$NODD_{DC}$	1mM PHD2-QM1-R396T + 1.2mM $MnCl_2$ + 2mM 2OG + 2mM $NODD_{DC}$	0.1 M citrate pH 5.0, 20 % w/v polyethylene glycol 6000	Sitting drop (300 nl), protein-to-well ratio, 1:1, 293K
PHD2-P317R•FG2216	1mM PHD2-P317R + 1.2mM $MnCl_2$ + 2mM FG2216	0.1 M tri-sodium citrate pH 5.6, 20% PEG 4000, 20% 2-propanol	Sitting drop (300 nl), protein-to-well ratio, 1:1, 293K
PHD2-R371H•FG2216	1mM PHD2-R371H + 1.2mM $MnCl_2$ + 2mM FG2216	0.1 M MES-Na pH 6.5, 30% polyethylene glycol monomethyl ether 5000, 0.2 M ammonium sulphate	Sitting drop (300 nl), protein-to-well ratio, 1:1, 293K
PHD2-R396T•FG2216	1mM PHD2-R396T + 1.2mM $MnCl_2$ + 2mM FG2216	0.1 M MES pH 6.5, 2.1 M ammonium sulphate, 2% v/v dioxane, 0.002 M $MnCl_2$	Sitting drop (300 nl), protein-to-well ratio, 1:2, 293K
PHD2- I280V/R281L/I292V •FG2216	1mM PHD2-I280V/R281L/I292V + 1.2mM $MnCl_2$ + 2mM FG2216	0.1 M MES pH 6.5, 1.8 M ammonium sulphate, 5% v/v dioxane, 0.002 M $MnCl_2$	Sitting drop (300 nl), protein-to-well ratio, 2:1, 293K
PHD2-G294E•FG2216	1mM PHD2-G294E + 1.2mM $MnCl_2$ + 2mM FG2216	0.1 M MES pH 6.5, 2.0 M ammonium sulphate, 7% v/v dioxane, 0.002 M $MnCl_2$	Sitting drop (300 nl), protein-to-well ratio, 2:1, 293K
PHD2-K293K/G294E•FG2216	1mM PHD2-K293K/G294E + 1.2mM $MnCl_2$ + 2mM FG2216	0.1 M MES pH 6.5, 1.8 M ammonium sulphate, 2% v/v dioxane, 0.002 M $MnCl_2$	Sitting drop (300 nl), protein-to-well ratio, 1:1, 293K

$^{\psi}$ Substrate Peptides used,

NODD: ^{395} DALTLLAPAAGDTIISLDF^{413};
$NODD_{DC}$ (L397C/D412C): ^{395}DA<u>C</u>TLLAPAAGDTIISL<u>C</u>F^{413};
CODD: ^{556}DLDLEMLAPYIPMDDDFQL^{574};

#PHD2 variants used for cross-linking,

PHD2-QM1: PHD2 C201A/R281C/P317C/R398A
PHD2-QM2: PHD2 C201A/R281C/V314C/R398A
PHD2-QM1-R396T: PHD2 C201A/R281C/P317C/R396T/R398A

PHD2 buffer used: 50 mM Tris pH 7.5, 1% (v/v) glycerol.

Supplementary Table 6 | Data collection and refinement statistics of PHD2.NOG and PHD2.ODD complexes.

	PHD2.Mn(II).NOG	PHD2.Mn(II).2OG. CODD†	PHD2-QM2.Mn(II). NOG. NODD	PHD2-QM1.Mn(II). NOG.NODD	PHD2QM1-R396T. Mn(II).NOG.NODD
**PDB acquisition codes**	5L9R	5L9B	5LA9	5L9V	5LAS
**Data collection**					
Beamline (Wavelength, Å)	DLS I02 (0.9795)	DLS I03 (0.9795)	DLS I04 (0.9795)	DLS I04 (0.9795)	DLS I04 (0.8344)
Detector	PILATUS 6M-F	PILATUS3 6M	ADSC Q315R	ADSC Q315R	PILATUS 6M-F
Data processing	HKL2000[22]	HKL2000[22]	HKL2000[22]	HKL2000[22]	HKL2000[22]
Space group	$P4_1$	$P2_1$	$P2_12_12$	$P2_1$	$P2_1$
Cell dimensions					
a, b, c (Å)	71.23, 71.23, 48.26	40.19, 76.39, 70.96	88.47, 97.33, 71.0	43.81, 73.09, 70.41	43.58, 73.64, 70.15
α, β, γ (°)	90, 90, 90	90, 90.03, 90	90, 90, 90	90, 91.17, 90	90, 91.25, 90
No. of molecules/ ASU	1	2	2	2	2
No. reflections	22342 (2211)*	31425 (3125)*	15152 (1486)*	38965 (3898)*	26258 (2580)*
Resolution (Å)	39.95-1.81 (1.87-1.81)*	40.19-1.95 (2.02-1.95)*	48.13-2.81 (2.90-2.81)*	43.80-1.83 (1.90-1.83)*	43.57-2.10 (2.18-2.10)*
R_{sym} or R_{merge}**	0.106 (0.977)*	0.167 (0.900)*	0.245 (1.250)*	0.101 (0.908)*	0.175 (0.681)*
$I/\sigma I$	18.4 (1.9)*	8.7 (1.7)*	4.8 (1.7)*	11.3 (2.0)*	7.8 (2.5)*
Completeness (%)	99.9 (99.1)*	99.1 (98.2)*	98.5 (99.3)*	99.7 (100)*	99.2 (98.8)*
Redundancy	7.3 (6.7)*	4.8 (4.7)*	3.4 (3.5)*	3.6 (3.7)*	3.4 (3.2)*
CC (1/2)	0.995 (0.722)*	0.998 (0.506)*	0.993 (0.510)*	0.993 (0.705)*	0.982 (0.641)*
Wilson B value (Å^2)	29.2	20.5	38.0	24.5	25.9
**Refinement**					
$R_{work/}$ R_{free}‡	0.158/0.188	0.157/0.187	0.251/0.279	0.169/0.190	0.185/0.221
No. atoms$^\psi$					
-Enzyme (A/B)	1730	1695/ 1681	1554/ 1615	1675/ 1682	1647/ 1615
-Metal (A/B)	1	1/ 1	1/ 1	1/ 1	1/ 1
-Ligand (A/B)	10 (NOG)	10/ 10 (2OG)	10/ 10 (NOG)	10/ 10 (NOG)	10/ 10 (NOG)
-Substrate (C/D)	-	145/ 146	130/ 130	131/ 131	131/ 131
-Water	132	298	74	224	205
B-factors$^\psi$					
-Enzyme (A/B)	38.0	24.7/ 25.2	39.4/ 38.3	35.5/ 38.3	42.2/ 46.6
-Metal (A/B)	22.4	18.4/ 13.5	18.2/ 17.9	21.1/ 15.6	28.7/ 30.6
-Ligand (A/B)	25.4	13.1/ 17.3	26.2/ 33.7	25.6/ 26.1	30.2/ 33.1
-Substrate (C/D)	-	30.3/ 32.7	45.2/ 38.7	35.6/ 38.1	42.9/ 48.3
-Water	49.8	32.7	29.0	41.3	47.4
R.m.s deviations					
-Bond lengths (Å)	0.005	0.003	0.003	0.005	0.004
-Bond angles (º)	0.776	0.610	0.612	0.711	0.702

*Highest resolution shell shown in parenthesis.

**$R_{sym} = \sum|I-\langle I\rangle|/\sum I$, where I is the intensity of an individual measurement and $\langle I\rangle$ is the average intensity from multiple observations.

†Refinement target: TWIN_LSQ_F.

‡$R_{factor} = \sum_{hkl}||F_{obs}(hkl)| - k\,|F_{calc}(hkl)||/\sum_{hkl}|F_{obs}(hkl)|$ for the working set of reflections; R_{free} is the R_{factor} for ~5% of the reflections excluded from refinement.

$^\psi$Polypeptide chain in parenthesis.

Supplementary Table 7. Data collection and refinement statistics of PHD2 clinical variants.

	PHD2-P317R.Mn(II). FG2216	PHD2-R371H.Mn(II). FG2216	PHD2-R396T.Mn(II). FG2216
**PDB acquisition codes**	5LAT	5LB6	5LBB
**Data collection**			
Beamline (Wavelength, Å)	DLS I04 (0.9795)	DLS I04 (0.9795)	DLS I03 (0.9763)
Detector	ADSC Q315 3X3	ADSC Q315 3X3	PILATUS3 6M
Data processing	MOSFLM[23], SCALA[24]	MOSFLM[23], SCALA[24]	XDS[25], SCALA[24]
Space group	$P6_3$	$P6_3$	$P6_3$
Cell dimensions			
a, *b*, *c* (Å)	111.18, 111.18, 40.03	110.67, 110.67, 40.23	110.20, 110.20, 39.72
α, *β*, *γ* (°)	90, 90, 120	90, 90, 120	90, 90, 120
No. of molecules/ ASU	1	1	1
No. reflections	22463 (3254)*	31670 (4575)*	29903 (4323)*
Resolution (Å)	27.80-1.90 (2.00-1.90)*	36.40-1.70 (1.79-1.70)*	36.07-1.70 (1.79-1.70)*
R_{sym} or R_{merge}**	0.088 (0.354)*	0.087 (0.500)*	0.038 (0.701)*
$I/\sigma I$	9.1 (2.9)*	18.0 (3.8)*	14.5 (2.2)*
Completeness (%)	99.6 (99.8)*	100 (100)*	97.8 (97.6)*
Redundancy	2.8 (2.9)*	10.5 (10.7)*	3.3 (3.4)*
CC (1/2)	0.991 (0.824)*	0.998 (0.922)*	0.999 (0.686)*
Wilson *B* value ($Å^2$)	21.4	20.1	31.4
**Refinement**			
$R_{work/}$ R_{free}‡	0.150/0.166	0.145/0.168	0.152/0.178
No. atoms			
-Enzyme	1767	1792	1779
-Metal	1	1	1
-Ligand (FG2216)	19	19	19
-Water	186	122	173
B-factors			
-Enzyme	29.3	42.2	30.4
-Metal	13.4	23.4	13.1
-Ligand (FG2216)	15.2	25.4	14.9
-Water	45.7	47.7	40.4
R.m.s deviations			
-Bond lengths (Å)	0.006	0.012	0.017
-Bond angles (º)	0.852	1.215	1.451

*Highest resolution shell shown in parenthesis.
**$R_{sym} = \sum|I - \langle I \rangle| / \sum I$, where *I* is the intensity of an individual measurement and <*I*> is the average intensity from multiple observations.
‡$R_{factor} = \sum_{hkl}||F_{obs}(hkl)| - k\,|F_{calc}(hkl)|| / \sum_{hkl}|F_{obs}(hkl)|$ for the working set of reflections; R_{free} is the R_{factor} for ~5% of the reflections excluded from refinement.

Supplementary Table 8 | Data collection and refinement statistics of PHD2 ODD-selective variants.

	PHD2-I280V/I292V/R281L. Mn(II).FG2216	PHD2-N293K/G294E.Mn(II). FG2216	PHD2-G294E.Mn(II).FG2216
**PDB acquisition codes**	5LBC	5LBE	5LBF
**Data collection**			
Beamline (Wavelength, Å)	DLS I04-1 (0.9173)	DLS I04 (0.9795)	DLS I02 (0.9795)
Detector	PILATUS 2M	PILATUS 6M-F	PILATUS 6M-F
Data processing	HKL2000[22]	HKL2000[22]	HKL2000[22]
Space group	$P6_3$	$P6_3$	$P6_3$
Cell dimensions			
a, *b*, *c* (Å)	111.00, 111.00, 40.28	109.77, 109.77, 39.55	110.45, 110.45, 39.65
α, *β*, *γ* (°)	90, 90, 120	90, 90, 120	90, 90, 120
No. of molecules/ ASU	1	1	1
No. reflections	25773 (2524)*	21775 (2153)*	28119 (2793)*
Resolution (Å)	48.07-1.81 (1.87-1.81)*	47.53-1.90 (1.97-1.90)*	32.21-1.75 (1.81-1.75)*
R_{sym} or R_{merge}**	0.092 (0.729)*	0.083 (1.0)*	0.050 (0.957)*
$I/\sigma I$	17.1 (2.0)*	30.8 (2.5)*	25.1 (2.2)*
Completeness (%)	99.5 (98.5)*	100 (100)*	100 (100)*
Redundancy	5.3 (3.2)*	14.7 (14.9)*	6.5 (6.3)*
CC (1/2)	0.997 (0.572)*	0.998 (0.577)*	0.998 (0.670)*
Wilson *B* value ($Å^2$)	25.1	35.0	34.0
**Refinement**			
$R_{work/}$ R_{free}‡	0.158/0.181	0.160/0.185	0.164/0.177
No. atoms			
-Enzyme	1780	1804	1720
-Metal	1	1	1
-Ligand (FG2216)	19	19	19
-Water	138	132	103
B-factors			
-Enzyme	35.0	46.8	48.6
-Metal	16.8	26.7	27.2
-Ligand (FG2216)	23.0	29.6	29.6
-Water	44.1	49.0	51.7
R.m.s deviations			
-Bond lengths (Å)	0.006	0.008	0.005
-Bond angles (º)	0.950	0.947	0.813

*Highest resolution shell shown in parenthesis.
**$R_{sym} = \sum|I-\langle I\rangle|/\sum I$, where I is the intensity of an individual measurement and $\langle I\rangle$ is the average intensity from multiple observations.
‡$R_{factor} = \sum_{hkl}||F_{obs}(hkl)| - k\,|F_{calc}(hkl)||/\sum_{hkl}|F_{obs}(hkl)|$ for the working set of reflections; R_{free} is the R_{factor} for ~5% of the reflections excluded from refinement.

SUPPLEMENTARY REFERENCES

1 Larkin, M. A., Blackshields, G., Brown, N. P., Chenna, R., McGettigan, P. A., McWilliam, H., Valentin, F., Wallace, I. M., Wilm, A., Lopez, R., Thompson, J. D., Gibson, T. J. & Higgins, D. G. Clustal W and Clustal X version 2.0. *Bioinformatics* **23**, 2947-2948 (2007).

2 Chowdhury, R., McDonough, M. A., Mecinovic, J., Loenarz, C., Flashman, E., Hewitson, K. S., Domene, C. & Schofield, C. J. Structural basis for binding of hypoxia-inducible factor to the oxygen-sensing prolyl hydroxylases. *Structure* **17**, 981-989 (2009).

3 McDonough, M. A., Li, V., Flashman, E., Chowdhury, R., Mohr, C., Lienard, B. M., Zondlo, J., Oldham, N. J., Clifton, I. J., Lewis, J., McNeill, L. A., Kurzeja, R. J., Hewitson, K. S., Yang, E., Jordan, S., Syed, R. S. & Schofield, C. J. Cellular oxygen sensing: Crystal structure of hypoxia-inducible factor prolyl hydroxylase (PHD2). *Proc. Natl. Acad. Sci. U. S. A.* **103**, 9814-9819 (2006).

4 Bamford, S., Dawson, E., Forbes, S., Clements, J., Pettett, R., Dogan, A., Flanagan, A., Teague, J., Futreal, P. A., Stratton, M. R. & Wooster, R. The COSMIC (Catalogue of Somatic Mutations in Cancer) database and website. *Br. J. Cancer* **91**, 355-358 (2004).

5 Chowdhury, R., Sekirnik, R., Brissett, N. C., Krojer, T., Ho, C. H., Ng, S. S., Clifton, I. J., Ge, W., Kershaw, N. J., Fox, G. C., Muniz, J. R., Vollmar, M., Phillips, C., Pilka, E. S., Kavanagh, K. L., von Delft, F., Oppermann, U., McDonough, M. A., Doherty, A. J. & Schofield, C. J. Ribosomal oxygenases are structurally conserved from prokaryotes to humans. *Nature* **509**, 422-426 (2014).

6 Yang, C. G., Yi, C., Duguid, E. M., Sullivan, C. T., Jian, X., Rice, P. A. & He, C. Crystal structures of DNA/RNA repair enzymes AlkB and ABH2 bound to dsDNA. *Nature* **452**, 961-965 (2008).

7 Mecinovic, J., Chowdhury, R., Flashman, E. & Schofield, C. J. Use of mass spectrometry to probe the nucleophilicity of cysteinyl residues of prolyl hydroxylase domain 2. *Anal. Biochem.* **393**, 215-221 (2009).

8 Appelhoff, R. J., Tian, Y. M., Raval, R. R., Turley, H., Harris, A. L., Pugh, C. W., Ratcliffe, P. J. & Gleadle, J. M. Differential function of the prolyl hydroxylases PHD1, PHD2, and PHD3 in the regulation of hypoxia-inducible factor. *J. Biol. Chem.* **279**, 38458-38465 (2004).

9 Flashman, E., Bagg, E. A., Chowdhury, R., Mecinovic, J., Loenarz, C., McDonough, M. A., Hewitson, K. S. & Schofield, C. J. Kinetic rationale for selectivity toward *N*- and *C*-terminal oxygen-dependent degradation domain substrates mediated by a loop region of hypoxia-inducible factor prolyl hydroxylases. *J. Biol. Chem.* **283**, 3808-3815 (2008).

10 Villar, D., Vara-Vega, A., Landazuri, M. O. & Del Peso, L. Identification of a region on hypoxia-inducible-factor prolyl 4-hydroxylases that determines their specificity for the oxygen degradation domains. *Biochem. J.* **408**, 231-240 (2007).

11 Masson, N., Willam, C., Maxwell, P. H., Pugh, C. W. & Ratcliffe, P. J. Independent function of two destruction domains in hypoxia-inducible factor-alpha chains activated by prolyl hydroxylation. *EMBO J.* **20**, 5197-5206 (2001).

12 Percy, M. J., Zhao, Q., Flores, A., Harrison, C., Lappin, T. R., Maxwell, P. H., McMullin, M. F. & Lee, F. S. A family with erythrocytosis establishes a role for prolyl hydroxylase domain protein 2 in oxygen homeostasis. *Proc. Natl. Acad. Sci. U. S. A.* **103**, 654-659 (2006).

13 Kageyama, Y., Koshiji, M., To, K. K., Tian, Y. M., Ratcliffe, P. J. & Huang, L. E. Leu-574 of human HIF-1α is a molecular determinant of prolyl hydroxylation. *Faseb J.* **18**, 1028-1030 (2004).

14 Landazuri, M. O., Vara-Vega, A., Viton, M., Cuevas, Y. & del Peso, L. Analysis of HIF-prolyl hydroxylases binding to substrates. *Biochem. Biophys. Res. Commun.* **351**, 313-320 (2006).

15 Rosen, M. D., Venkatesan, H., Peltier, H. M., Bembenek, S. D., Kanelakis, K. C., Zhao, L. X., Leonard, B. E., Hocutt, F. M., Wu, X., Palomino, H. L., Brondstetter, T. I., Haugh, P. V., Cagnon, L., Yan, W., Liotta, L. A., Young, A., Mirzadegan, T., Shankley, N. P., Barrett, T. D. & Rabinowitz, M. H. Benzimidazole-2-pyrazole HIF Prolyl 4-Hydroxylase Inhibitors as Oral Erythropoietin Secretagogues. *ACS Med. Chem. Lett.* **1**, 526-529 (2010).

16 Na, Y. R., Woo, D. J., Choo, H., Chung, H. S. & Yang, E. G. Selective inhibition of the hypoxia-inducible factor prolyl hydroxylase PHD3 by Zn(II). *Chem. Commun.* **51**, 10730-10733 (2015).

17 Arsenault, P. R., Pei, F., Lee, R., Kerestes, H., Percy, M. J., Keith, B., Simon, M. C., Lappin, T. R., Khurana, T. S. & Lee, F. S. A knock-in mouse model of human PHD2 gene-associated erythrocytosis establishes a haploinsufficiency mechanism. *J. Biol. Chem.* **288**, 33571-33584 (2013).

18 Percy, M. J., Furlow, P. W., Beer, P. A., Lappin, T. R., McMullin, M. F. & Lee, F. S. A novel erythrocytosis-associated PHD2 mutation suggests the location of a HIF binding groove. *Blood* **110**, 2193-2196 (2007).

19 van der Donk, W. A., Krebs, C. & Bollinger, J. M., Jr. Substrate activation by iron superoxo intermediates. *Current opinion in structural biology* **20**, 673-683 (2010).

20 West, C. M. & Blader, I. J. Oxygen sensing by protozoans: how they catch their breath. *Curr. Opin. Microbiol.* **26**, 41-47 (2015).

21 Vranken, W. F., Boucher, W., Stevens, T. J., Fogh, R. H., Pajon, A., Llinas, M., Ulrich, E. L., Markley, J. L., Ionides, J. & Laue, E. D. The CCPN data model for NMR spectroscopy: development of a software pipeline. *Proteins* **59**, 687-696 (2005).

22 Otwinowski, Z. & Minor, W. Processing of X-ray diffraction data collected in oscillation mode. *Method Enzymol.* **276**, 307-326 (1997).

23 Battye, T. G., Kontogiannis, L., Johnson, O., Powell, H. R. & Leslie, A. G. iMOSFLM: a new graphical interface for diffraction-image processing with MOSFLM. *Acta Crystallogr. D Biol. Crystallogr.* **67**, 271-281 (2011).

24 Winn, M. D., Ballard, C. C., Cowtan, K. D., Dodson, E. J., Emsley, P., Evans, P. R., Keegan, R. M., Krissinel, E. B., Leslie, A. G., McCoy, A., McNicholas, S. J., Murshudov, G. N., Pannu, N. S., Potterton, E. A., Powell, H. R., Read, R. J., Vagin, A. & Wilson, K. S. Overview of the CCP4 suite and current developments. *Acta Crystallogr. D Biol. Crystallogr.* **67**, 235-242 (2011).

25 Kabsch, W. Integration, scaling, space-group assignment and post-refinement. *Acta Crystallogr. D Biol. Crystallogr.* **66**, 133-144 (2010).
